# Infiltrating Blood-Derived Macrophages Are Vital Cells Playing an Anti-inflammatory Role in Recovery from Spinal Cord Injury in Mice

**DOI:** 10.1371/journal.pmed.1000113

**Published:** 2009-07-28

**Authors:** Ravid Shechter, Anat London, Chen Varol, Catarina Raposo, Melania Cusimano, Gili Yovel, Asya Rolls, Matthias Mack, Stefano Pluchino, Gianvito Martino, Steffen Jung, Michal Schwartz

**Affiliations:** 1 Department of Neurobiology, The Weizmann Institute of Science, Rehovot, Israel; 2 Department of Immunology, The Weizmann Institute of Science, Rehovot, Israel; 3 Neuroimmunology Unit, DIBIT and Institute of Experimental Neurology (INSPE), San Raffaele Scientific Institute, Milan, Italy; 4 Department of Internal Medicine, University of Regensburg, Regensburg, Germany; Imperial College London, United Kingdom

## Abstract

**Background:**

Although macrophages (MΦ) are known as essential players in wound healing, their contribution to recovery from spinal cord injury (SCI) is a subject of debate. The difficulties in distinguishing between different MΦ subpopulations at the lesion site have further contributed to the controversy and led to the common view of MΦ as functionally homogenous. Given the massive accumulation in the injured spinal cord of activated resident microglia, which are the native immune occupants of the central nervous system (CNS), the recruitment of additional infiltrating monocytes from the peripheral blood seems puzzling. A key question that remains is whether the infiltrating monocyte-derived MΦ contribute to repair, or represent an unavoidable detrimental response. The hypothesis of the current study is that a specific population of infiltrating monocyte-derived MΦ is functionally distinct from the inflammatory resident microglia and is essential for recovery from SCI.

**Methods and Findings:**

We inflicted SCI in adult mice, and tested the effect of infiltrating monocyte-derived MΦ on the recovery process. Adoptive transfer experiments and bone marrow chimeras were used to functionally distinguish between the resident microglia and the infiltrating monocyte-derived MΦ. We followed the infiltration of the monocyte-derived MΦ to the injured site and characterized their spatial distribution and phenotype. Increasing the naïve monocyte pool by either adoptive transfer or CNS-specific vaccination resulted in a higher number of spontaneously recruited cells and improved recovery. Selective ablation of infiltrating monocyte-derived MΦ following SCI while sparing the resident microglia, using either antibody-mediated depletion or conditional ablation by diphtheria toxin, impaired recovery. Reconstitution of the peripheral blood with monocytes resistant to ablation restored the lost motor functions. Importantly, the infiltrating monocyte-derived MΦ displayed a local anti-inflammatory beneficial role, which was critically dependent upon their expression of interleukin 10.

**Conclusions:**

The results of this study attribute a novel anti-inflammatory role to a unique subset of infiltrating monocyte-derived MΦ in SCI recovery, which cannot be provided by the activated resident microglia. According to our results, limited recovery following SCI can be attributed in part to the inadequate, untimely, spontaneous recruitment of monocytes. This process is amenable to boosting either by active vaccination with a myelin-derived altered peptide ligand, which indicates involvement of adaptive immunity in monocyte recruitment, or by augmenting the naïve monocyte pool in the peripheral blood. Thus, our study sheds new light on the long-held debate regarding the contribution of MΦ to recovery from CNS injuries, and has potentially far-reaching therapeutic implications.

*Please see later in the article for Editors' Summary*

## Abstract 2

Using a mouse model of spinal injury, Michal Schwartz and colleagues tested the effect of macrophages on the recovery process and demonstrate an important anti-inflammatory role for a subset of infiltrating monocyte-derived macrophages that is dependent upon their expression of interleukin 10.

## Editors' Summary

**Background:**

Every year, spinal cord injuries paralyze about 11,000 people in the US. The spinal cord, which contains bundles of nervous system cells called neurons, is the communication highway between the brain and the body. Messages from the brain travel down the spinal cord to control movement, breathing and other bodily functions; messages from the skin and other sensory organs travel up the spinal cord to keep the brain informed about the body. The bones of the spine normally protect the spinal cord but, if these are broken or displaced, the spinal cord can be cut or compressed, which interrupts the information flow. Damage near the top of the spinal cord paralyzes the arms and legs (tetraplegia); damage lower down paralyzes the legs only (paraplegia). Spinal cord injuries also cause other medical problems, including the loss of bladder and bowel control. Currently, there is no effective treatment for spinal cord injuries, which usually cause permanent disability because the damaged nerve fibers rarely regrow.

**Why Was This Study Done?:**

After a spinal cord injury, immune system cells called macrophages accumulate at the injury site. Some of these macrophages—so-called monocyte-derived macrophages—come into (infiltrate) the spinal cord from the blood in response to the injury, whereas others—microglia—are always in the nervous system. Although macrophages are essential for wound healing in other parts of the body, it is unclear whether they have good or bad effects in the spinal cord. Many experts believe that immune system cells hinder healing in the spinal cord and should be suppressed or eliminated, but other scientists claim that macrophages secrete factors that stimulate nerve regrowth. Furthermore, although some macrophages elsewhere in the body have proinflammatory (potentially deleterious) effects, others have anti-inflammatory (beneficial) effects. So do the infiltrating monocyte-derived macrophages and the resident microglia (which are proinflammatory) have different functions at spinal cord injury sites? In this study, the researchers try to answer this important question.

**What Did the Researchers Do and Find?:**

The researchers bruised a small section of the spinal cord of adult mice and then investigated the effect of infiltrating monocyte-derived macrophages on the recovery process. Monocyte-derived macrophages and microglia cannot be distinguished using standard staining techniques so to study their behavior after spinal cord injury the researchers introduced labeled monocyte-derived macrophages into their experimental animals by using adoptive transfer (injection of genetically labeled monocytes into the animals) or by making bone marrow chimeras. In this second technique, the animals' monocyte-derived macrophages (but not their microglia) were killed by irradiating the animals before injection of genetically labeled bone marrow, the source of monocytes. Using these approaches, the researchers found that monocyte-derived macrophages collected at the margins of spinal cord injury sites whereas microglia accumulated throughout the sites. When the pool of monocyte-derived macrophages in the mice was increased by adoptive transfer or by using a technique called “CNS-specific vaccination,” more monocyte-derived macrophages infiltrated the injury site and the animals' physical recovery from injury improved. Conversely, removal of the infiltrating monocyte-derived macrophages from the injury site reduced the animals' physical recovery. Other experiments indicated that the infiltrating monocyte-derived macrophages have a beneficial, local anti-inflammatory effect that is dependent on their expression of interleukin-10 (an anti-inflammatory signaling molecule).

**What Do These Findings Mean?:**

These findings provide new information about the contribution of monocyte-derived macrophages to spontaneous recovery from spinal cord injury, a contribution that has long been debated. In particular, the findings suggest that this subset of macrophages (but not the resident microglia) has a beneficial effect on spinal cord injuries that is mediated by their production of the anti-inflammatory molecule interleukin-10. The findings also show that the effect of these monocyte-derived macrophages can be boosted, at least in mice. Although results obtained in experiments done in animals do not always accurately reflect what happens in people, this new understanding of the different functions of microglia and infiltrating monocyte-derived macrophages after injury to the spinal cord may eventually lead to the development of better treatments for spinal cord injuries.

**Additional Information:**

Please access these Web sites via the online version of this summary at http://dx.doi.org/10.1371/journal.pmed.1000113.

- The MedlinePlus encyclopedia provides information about spinal cord injuries (in English and Spanish)
- The US National Institute of Neurological Disorders and Stroke provides detailed information about spinal cord injury, including information on current research into the problem (in English and Spanish)
- MedlinePlus provides an interactive tutorial on spinal cord injury and a list of links to additional information (in English and Spanish)

## Introduction

Immune cells play a critical role in the resolution of wound healing and pathologies that occur in peripheral organs. However, in the central nervous system (CNS), which is considered an immune-privileged site, the contribution of these cells to the healing process remains a subject of controversy [1]–[14].

Following CNS injury, an intensive local inflammatory response takes place and involves activated resident microglia, the native macrophages (MΦ) of the CNS, and an additional population of MΦ that derives from monocytes which infiltrate to the CNS from the peripheral blood only following the insult [15]; in this study, we refer to this blood-derived population as monocyte-derived MΦ. These two MΦ populations are indistinguishable by standard immunohistochemical techniques leading to the common view of MΦ as functionally homogenous. The limited spontaneous recovery, along with the common negative perception of the local inflammation, have led to the general view of all immune cells at the lesion site as destructive cells that should be suppressed or eliminated [1],[2],[6],[7],[10],[16]–[19]. Accordingly, research efforts and clinical manipulations were directed at attempts to overcome this perceived obstacle to recovery [20], through the use of high-dose steroids, and by nonspecific MΦ depletion [1],[21]. However, other studies have demonstrated that “alternatively activated MΦ,” pre-incubated *ex-vivo* with peripheral nerve segments, can induce CNS repair [22]. Moreover, several independent studies have demonstrated that MΦ are capable of secreting neurotrophic factors [23]–[25], can promote removal of tissue debris [26],[27], and can support axonal regeneration [28]–[33].

Outside the CNS, it has recently been recognized that MΦ represent a heterogeneous population that can exhibit both pro- and anti-inflammatory activities [34]–[37]. Altogether, the apparent contradictory data regarding the contribution of MΦ to CNS recovery, and the recognized heterogeneity of MΦ outside the CNS, have led us to suggest that a similar scenario may apply to the CNS, and that perhaps infiltrating monocyte-derived MΦ have a unique role that is not performed by the well-described pro-inflammatory resident microglia.

Here, we investigated the specific contribution of the monocyte-derived MΦ recruited to the damaged tissue.

## Methods

### Animals

Seven types of mice were used: (1) C57BL/6J mice (*n* = 374 and another 22 as donors of monocytes); (2) heterozygous mutant *Cx_3_cr1* ^GFP/+^ mice (B6.129P- *Cx_3_cr1*tm1Litt/J, in which one of the *CX_3_CR1* chemokine receptor alleles is replaced with a gene encoding GFP [green fluorescent protein] [38]; *n* = 12 as donors of monocytes, and another eight as donors of bone marrow [BM]); (3) CD45.1 mice (carrying an allotypic marker, CD45.1; *n* = 8, another 24 as donors of monocytes, and another three as donors of BM); (4) *Cx_3_cr1* ^GFP/+^(CD45.1) transgenic mice (carrying both the *Cx_3_cr1* ^GFP^ locus and the allotypic marker, CD45.1; *n* = 8 as donors of monocytes, and another one as a donor of BM); (5) *CD11c*-DTR transgenic mice (B6.FVB-Tg Itgax-DTR/GFP 57Lan/J [39], carrying a transgene encoding a human diphtheria toxin receptor [DTR] under control of the murine *CD11c* promoter; *n* = 10 as donors of BM); (6) *CD11c*-DTR: *Cx_3_cr1* ^GFP/+^ transgenic mice (heterozygous for both the *Cx_3_cr1* ^GFP^ locus and the *CD11c*-DTR transgene; *n* = 3 as donors of BM); and (7) IL-10 null mice (B6.129P2-Il10tm1Cgn/J [40]; *n* = 40 as donors of monocytes; a generous gift from Prof. Irun Cohen, Weizmann Institute of Science). In each case, adult males aged 8–10 wk were used. Animals were supplied by the Animal Breeding Center of The Weizmann Institute of Science. All animals were handled according to the regulations formulated by the Institutional Animal Care and Use Committee (IACUC).

### BM Radiation Chimeras

[*Cx_3_cr1* ^GFP/+^>wt], [*CD11c*-DTR>wt], [CD45.1>wt (CD45.2)], and [*CD11c*-DTR: *Cx_3_cr1* ^GFP/+^>wt] BM chimeras were prepared by subjecting gender-matched recipient mice (8–10 wk old) to lethal whole-body irradiation (950 rad) while shielding the brain, as previously described [41]–[43]. Empirically, we found that this shielding prevented the massive infiltration of myeloid cells to noninjured spinal cords (Figure S1A, S1B). Following SCI, however, an intense infiltration of GFP^+^ cells was seen in the spinal cord of these head-shielded chimeras (Figure S1C, S1D). The mice were then reconstituted with 3–5×10^6^ BM cells harvested from the hind limbs (tibia and femur) and forelimbs (humerus) of the appropriate donor mice. BM cells were obtained by flushing the bones with Dulbecco's PBS under aseptic conditions, and then they were collected and washed by centrifugation (10 min, 1,000 rpm, 4°C). The extent of chimerism observed in these chimeras (68.5%±2.2%; [mean±standard error (SE)]) was comparable to that reported elsewhere [8],[44],[45]. To rule out the possibility that the observed homing of monocytes to the injured site of the chimeric mice was due to any side effect related to the process of the chimerism, the homing of grafted monocytes to the injured site in chimeric versus nonchimeric mice was compared; no differences were found (Figure S1E). Thus, the use of chimeras created with head shielding preserved the physiological recruitment and homing of MΦ following SCI. The chimeric mice were subjected to spinal cord contusion 8–10 wk after BM transplantation.

### Antigens and Vaccination

A MOG-derived altered peptide ligand, MEVGWYRSPFDRVVHLYRNGK (MOG-45D) (an analog of pMOG 35–55), in which aspartic acid is substituted for serine [46], was prepared by the Peptide Synthesis Unit at the Weizmann Institute. 45D peptide is a weak agonist of the encephalitogenic MOG 35–55 peptide and does not induce autoimmune disease [46]. Ovalbumin (OVA) peptide was purchased from Sigma-Aldrich (Rehovot, Israel). Adult mice were vaccinated with 45D or OVA (100 µg) emulsified in an equal volume of complete Freund's adjuvant (CFA; Difco) containing *Mycobacterium tuberculosis* (2.5 mg/ml; Difco), as previously described [47],[48]. The emulsion (total volume 0.1 ml) was injected subcutaneously at one site in the flank, 7 d prior to the spinal cord injury.

### Spinal Cord Injury

The spinal cords of deeply anesthetized mice were exposed by laminectomy at T12, and contusive (200 kdynes) centralized injury was performed using the Infinite Horizon spinal cord impactor (Precision Systems), as previously described [47],[48] causing bilateral degeneration without complete penetration of the spinal cord. The animals were maintained on twice-daily bladder expression. Animals that were contused in a nonsymmetrical manner were excluded from the experimental analysis.

### Assessment of Functional Recovery from Spinal Cord Contusion

Recovery was evaluated by hind-limb locomotor performance, assessed according to the open-field Basso Mouse Scale (BMS) [49], with nonlinear scores ranging from 0 (complete paralysis) to 9 (normal mobility); each score represents a distinct motor functional state. We randomly separated the mice into groups without any preferences, while verifying that the average starting score was similar in all groups. Blind scoring ensured that observers were not aware of the treatment received by each mouse. Locomotor activity in an open field was monitored twice a week by placing the mouse for 4 min at the center of a circular enclosure (diameter 90 cm, wall height 7 cm) made of molded plastic with a smooth, nonslippery floor. Before each evaluation, the mice were carefully examined for peritoneal infection, wounds in the hind limbs, and tail and foot autophagia. Animals that showed a difference of more than 2 score points between their two hind limbs were excluded from the experimental analysis. The results showing functional outcomes presented in this study were, in each case, from a single experiment representative of several independent replicates, as indicated in the figure legends.

### Diphtheria Toxin Administration

Diphtheria toxin (DTx; 8 ng/g body weight; Sigma) was injected intraperitoneally (IP), repeatedly at 1-d intervals, starting immediately after the injury, unless described otherwise. The efficiency of DTx treatment was routinely confirmed both in the periphery, by assessing the ablation of CD11c^hi^ cells in the spleen (Figure S2), and at the lesion site.

### MC-21 Administration

MC-21 (an antibody to CCR2) [50] was injected IP starting immediately after the injury throughout the first week of recovery (d0, d1, d2, d4, and d6 postinjury).

### Adoptive Monocyte Transfer

Gr1^+^ monocytes were isolated as previously reported [51]. Briefly, BM cells were harvested from the femora and tibiae of naïve mice, and enriched for mononuclear cells on a Ficoll density gradient. The CD115^+^ BM monocyte population was isolated through MACS enrichment using biotinylated anti-CD115 antibodies and streptavidin-coupled magnetic beads (Miltenyi Biotec) according to the manufacturers' protocols. Following this procedure, monocytes (wt, *Cx_3_cr1* ^GFP/+^ [CD45.1] or IL-10 deficient; purity 90%) were intravenously (IV) injected (3.5×10^6^ cells per mouse) during the first week of recovery (two or three injections, as indicated in the figures). This procedure theoretically enriched the subset of “Gr1^+^ monocytes” (commonly recruited to inflamed tissues) in the peripheral blood by 3-fold, as their total numbers in the circulation is approximately 10^6^ cells.

### Immunohistochemistry

Due to technical limitations of some of the antibodies that were used, two different tissue preparation protocols (paraffin embedded and microtomed frozen sections) were applied, as previously described [41]. Whenever possible, the results were confirmed using both techniques. The following antibodies were used: rabbit anti-GFP (1∶100; MBL); rabbit anti- glial fibrillary acidic protein (GFAP; 1∶100; Dako Cytomation), goat anti-IL-10 (1∶20; R&D Systems), mouse anti-arginase I (1∶100; BD Biosciences), rat anti-Ly6C (1∶200; Abcam), and hamster anti-CD11c (1∶50; Chemicon). For microglial/ MΦ labeling, TRITC- or FITC-conjugated *Bandeiraea simplicifolia* isolectin B4 (IB-4; 1∶50; Sigma-Aldrich) was added for 1 h to the secondary antibody solution. Secondary antibodies used included: Cy2-conjugated donkey anti-rabbit antibody, Cy2/Cy5 conjugated donkey anti-mouse antibody, Cy3-conjugated donkey anti-mouse, Cy3-conjugated donkey anti-goat, and biotin goat anti-hamster (1∶200; all from Jackson Immuno Research). Cy3-streptavidin was used for CD11c staining. The slides were exposed to Hoechst stain (1∶4,000; Invitrogen Probes) for 1 min.

Myelin integrity was qualitatively examined on paraffin-embedded sections that were stained with Luxol fast blue for myelin, and with Nissl to identify the nuclei and the thin cytoplasmic layer around them. GFAP staining was used for demarcation of the lesion site.

### Isolation of Spinal Cord Cells and Flow Cytometric Analysis

Mice subjected to spinal cord injury were killed by an overdose of anaesthetic and their spinal cords were prepared for flow cytometric analysis by perfusion with PBS via the left ventricle. Spinal cord sections were cut from individual mice, including the injured site and adjacent margins, or an area distal to it (4 mm long in each of the sections), and tissues were homogenized using a software controlled sealed homogenization system (Dispomix; http://www.biocellisolation.com). The following fluorochrome-labeled monoclonal antibodies were purchased from BD Pharmingen, BioLegend, or eBioscience and used according to the manufacturers' protocols: PE conjugated anti-CD11c, MHCII, Gr1(Ly6C), CD34, Mac3, CD11b, CD31, CD4, TCRβ, and CD115 antibodies; allophycocyanin-conjugated anti-CD45.1, Gr1 (Ly6C/G), and CD8 antibodies; AlexaFluor anti-CD19 antibodies; PerCP-conjugated anti-CD11b antibody; and biotin-conjugated anti-CD115 antibody. Cells were analyzed on a FACSCalibur cytometer (BD Biosciences) using CellQuest software (BD Biosciences). Isotype controls were routinely used in all the experiments. In addition, in each experiment, relevant negative control groups were used to determine the populations of interest and to exclude others.

### Data Quantification

For microscopic analysis, a Nikon fluorescent microscope (Nikon E800) or Zeiss LSM 510 confocal laser scanning microscope were used. Longitudinal sections of the spinal cord were analyzed. Numbers of cells, immunoreactivity (density) and lesion size were determined automatically with Image-Pro Plus 4.5 software (Media Cybernetics). For quantification of number of cells, a unique quantification method was used, based on Image-Pro software, that takes into consideration the accumulation of cells in clusters. Manual quantification and flow-cytometric analysis were also performed to verify the results provided by the automated analysis. In order to avoid overestimation due to counting of partial cells that appeared within the section, we took special care to count only cells with intact morphology and a nucleus that was larger than 4 µm in diameter, both in the manual and software-automated counting. Throughout the study, the area counted was 1–2 mm^2^. The number of cells per mm^3^ was calculated by considering the thickness of the sections. For quantification based on FACS analysis (*n* = 4–6 mice per group), positive events were counted in 25% of the sample, except for the adoptive transfer experiments in which the entire sample was analyzed.

### Statistical Analysis

Data were analyzed using the Student's *t*-test to compare between two groups. One-way or two-way ANOVA was used to compare several groups; the Tukey's HSD procedure (*p* = 0.05) was used for follow-up pairwise comparison of groups. Repeated measures ANOVA was used in the functional BMS scoring with follow-up comparison of treatments for each day by contrast *t*-test and correction for multiple comparison by the Holm method (*p* = 0.05). The specific tests used to analyze each set of experiments are indicated in the figure legends. The results are presented as mean±SE. In the graphs, *y*-axis error bars represent SE.

## Results

### Macrophages Derived from Infiltrating Monocytes Are Recruited to the Margins of the Lesion Site Following Spinal Cord Injury

As circulating precursor cells, monocytes are well known to infiltrate sites of injury outside of and within the CNS [9],[36],[37]. To test their involvement in the recovery from SCI, we first performed an adoptive transfer of labeled naïve monocytes to investigate features of their recruitment and fate. The monocyte graft was isolated from *Cx_3_cr1* ^GFP/+^ mice [38] (C57BL/6, CD45.1) that carry a genetic reporter gene label (*Cx_3_cr1* ^GFP/+^) and an allotypic marker (CD45.1), allowing detection of graft-derived cells in the recipient animals (C57BL/6, CD45.2) by immunohistochemistry and by flow cytometric analysis, respectively. Wild type (wt) C57BL/6 mice were subjected to a severe, well-calibrated contusive SCI at the level of T-12 [47]. Following injury, the animals were injected intravenously with labeled naïve monocytes (*Cx_3_cr1* ^GFP/+^/CD45.1^+^). As we did not yet know the time frame following injury at which the monocyte-derived MΦ infiltrate the CNS, and whether and when they are needed, we repeatedly injected naïve monocytes (3.5×10^6^ cells) during the first week after the injury (d0, d3 postinjury), thereby trying to ensure continuous and stable elevated levels of graft-derived naïve monocytes in the blood. This procedure theoretically enriches the subset of “Gr1^+^ monocytes” (commonly recruited to inflamed tissues) in the peripheral blood by 3-fold, as their numbers in the circulation is approximately 10^6^ cells.

**Figure 1:**
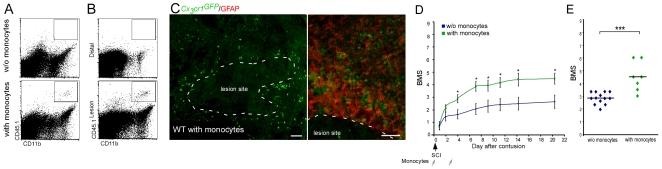
Monocyte-derived macrophages, spontaneously recruited to the injured spinal cord following the injury, promote functional recovery. Wild-type mice were subjected to SCI and received passive transfer (injected intravenously) of monocytes (CD45.1^+^ or *Cx_3_cr1* ^GFP^) during the first week of recovery. (A–C) Spinal cords were excised 7 d after the injury and analyzed for the presence of infiltrating monocyte-derived MΦ. Flow cytometric analysis of (A) lesion area (4 mm segment) of injured spinal cord from mice treated with and without (w/o) adoptive transfer of monocytes (CD45.1^+^/CD11b^+^), indicating the arrival of graft-derived MΦ to the lesion area. (B) Flow cytometric analysis of lesion and distal areas (4 mm segment each) from injured spinal cords of adoptively transferred mice indicating the accumulation of the graft-derived MΦ (CD45.1^+^/CD11b^+^) mainly at the lesion and not at the distal areas (2,259±431 engrafted cells per gram of tissue taken from lesion area [mean±SE]). (C) Immunohistochemical analysis showing the adoptively transferred cells (*Cx_3_cr1* ^GFP/+^; green) restricted to the margins of the lesion site, delineated by GFAP expression (red, right frame) (scale bar = 100 µm). (D) Similarly treated animals were followed for locomotor activity assessed according to the BMS (repeated measures ANOVA; F[between groups]_1,18_ = 16.7; *p* = 0.0007). *y*-Axis error bar represents SE. (E) Mean locomotor score (BMS) of individual mice on d28 after spinal cord injury (Student's *t*-test; *t* = −5.09; df = 15; *p* = 0.0001), suggesting that increasing the pool of naïve monocytes by IV injection of wt mice following SCI enhanced recovery beyond spontaneous levels. The assessment of the functional outcome presented here is from one experiment representative of three independent experiments performed.

Flow cytometric and histological analysis 7 d after transfer revealed the presence of labeled injected cells (GFP^+^, CD45.1^+^) at the lesion site, but not distal to it (Figure 1A–1C). Interestingly, these GFP^+^ monocyte-derived MΦ were concentrated at the margins of the lesion, but were absent from the epicenter (Figure 1C).

We next examined whether the augmentation of the physiological pool of naïve monocytes in the peripheral blood following SCI, by adoptive transfer of naïve monocytes, could enhance motor function recovery beyond the spontaneous level. Recovery of motor function was evaluated using a scale for hind limb motor ability in an open field (BMS [49]), in which a score of 0 indicates complete paralysis, and a score of 9 represents complete mobility. The augmentation of the monocyte pool resulted in recovery that exceeded the spontaneous recovery levels (Figure 1D, 1E; BMS score [mean±SE]: 4.5±0.4 and 3.0±0.1, respectively). These results suggested that monocytes can home from the peripheral blood to the margins of the lesion site following injury, and that increasing their numbers leads to improved recovery. However, these results were not sufficient to determine when the monocytes are needed, why this improved recovery does not occur spontaneously, and what the unique role of the monocyte-derived MΦ is that could not be provided by the resident microglia pre-existing in the injured site. To address these questions, we could not use this experimental paradigm of adoptive transfer of labeled monocytes to spinally injured wt mice; while the graft-derived MΦ could be discriminated from activated microglia according to their label (Figure 1), unlabeled infiltrating endogenous host monocyte-derived MΦ are indistinguishable from the resident microglia by standard immunohistochemical techniques. Thus, to differentiate between the resident activated microglia and the endogenously infiltrating monocyte-derived MΦ, we used an established BM chimera approach based on the unique radioresistance of resident microglia [9],[15],[26]. CX_3_CR1 is expressed on monocyte-derived MΦ and resident microglia, and therefore, in transgenic mice that carry a GFP gene under the control of the *CX_3_CR1* promoter, both of these populations are labeled [38],[52],[53]. However, in [*Cx_3_cr1* ^GFP/+^>wt] BM chimeras, whose wt BM was replaced with *Cx_3_cr1* ^GFP/+^ BM, the GFP-label is restricted to monocytes, thereby enabling infiltrating monocyte-derived MΦ to be distinguished from resident microglia [9]. Notably, preparation of the chimeras requires irradiation, which has been reported to induce immune cell recruitment to the CNS [8],[9]. Indeed, we observed in [*Cx_3_cr1* ^GFP/+^>wt] BM chimeras monocyte infiltration to both the brain (Butovsky et al., personal communication) and spinal cord, even in the absence of any injury (Figure S1A, S1B). Therefore, all the chimeric mice that were used in our study were prepared in a way that prevents irradiation-induced monocyte infiltration to the spinal cord in the absent of an insult (see Methods).

**Figure 2:**
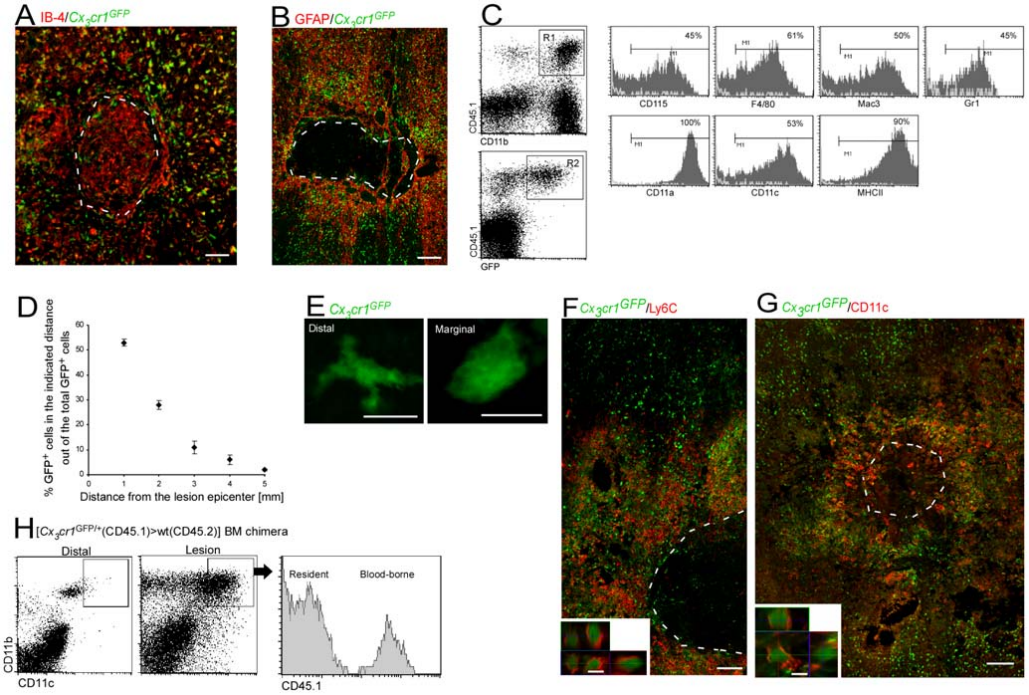
Monocyte-derived macrophages acquire a unique phenotype in close proximity to the lesion site. Chimeric mice were subjected to SCI and analyzed a week later for homing of cells. (A) Cells labeled for IB-4 (red), and GFP (green) at the lesion site of [*Cx_3_cr1* ^GFP/+^>wt] BM chimeric mice. (B) Cells labeled for GFAP (red) and GFP (green), demonstrating that the infiltrating myeloid cells barely penetrate the lesion epicenter. (C) Representative flow cytometric analysis showing the extent of expression of various markers by the infiltrating myeloid cells (CD11b^+^/ *Cx_3_cr1* ^GFP/+^/CD45.1^+^) in the injured spinal cords of [*Cx_3_cr1* ^GFP/+^ (CD45.1)>wt (CD45.2)] BM chimeras. The numbers above the bars refer to the percentage of the cells positive for the indicated marker out of the R1×R2 population (representing the infiltrating monocytes). The bars point to cells positive for the indicated marker (isotype control, gray line). (D) Spatial distribution map of monocyte-derived MΦ (GFP^+^) at specific distances relative to the epicenter of the lesion site in [*Cx_3_cr1* ^GFP/+^>wt] BM chimeras based on immunohistochemical analysis. (E) High magnification of GFP^+^ cells (green) from distal and marginal areas of the lesion, demonstrating morphological differences. (F) Representative confocal micrograph of longitudinal sections from injured spinal cord of [*Cx_3_cr1* ^GFP/+^>wt] BM chimeras, labeled for Ly6C (red), and infiltrating monocyte-derived MΦ by GFP (green). Lower panel: *z*-axis projection of a single cell. (G) Representative confocal micrograph of longitudinal sections of injured spinal cord, labeled for monocyte-derived MΦ by GFP (green) and CD11c (red). Lower panel: *z*-axis projection of single cell. (H) Flow cytometric analysis of distal and lesion spinal cord samples for CD11c expression on MΦ (CD11b+ cells) in [*Cx_3_cr1* ^GFP/+^(CD45.1)>wt(CD45.2)] BM chimeras. Note the higher incidence of CD11c^high^ cells in the lesion sample. The histogram to the right is gated on CD11b^+^/CD11c^high^ cells at the lesion area, showing that both resident (CD45.1^−^) and infiltrating (CD45.1^+^) cells express CD11c. The dashed line demarcates the lesion site in (A), (B), (F), and (G), as determined by GFAP immunoreactivity. Scale bar in (B) and (G), represents 250 µm; in (A) and (F), 100 µm; in (E), and lower panels of (F) and (G), 10 µm. Five mice were analyzed in each group.

Following the SCI, both resident microglia and the infiltrating monocyte-derived MΦ expressed the activation marker IB-4 (IB-4^+^/GFP^−^ versus IB-4^+^/GFP^+^ cells, respectively) (Figure 2A). Importantly, the spatial distribution of the infiltrating endogenous monocyte-derived MΦ in the parenchyma of the injured spinal cord of the chimeric mice was similar to that seen following the passive transfer of monocytes to nonchimeric injured mice (Figure 1C); monocyte-derived MΦ were restricted to the margins of the lesion site (demarcated by GFAP expression) and excluded from the epicenter (Figure 2A, 2B). These results supported the concept, demonstrated above (Figure S1), that the infiltration of monocyte-derived MΦ to the injured spinal cord in the chimeric mice is a physiological phenomenon, and enabled us to use this model for further investigation of the monocyte-derived MΦ distribution and contribution. Notably, monocyte-derived MΦ localization to the margins of the lesion appeared to be independent of the lesion severity (Figure S3). In addition, the chimeric mice enabled us to determine the spatial distribution of the resident microglia. Thus, the activated resident microglia (IB-4^+^/GFP^−^), unlike the infiltrating monocyte-derived MΦ, were ubiquitously distributed throughout the lesion center and at its margins (Figure 2A).

To gain further insight to the features of the infiltrating cells, we performed a comprehensive flow cytometric analysis. Because of the scarcity of the infiltrating cells, we adopted a new protocol that enabled efficient collection of the cells (see Methods for details). The bulk of infiltrating cells represented myeloid cells expressing CD11b, CD115, F4/80, Mac3, and Gr1 (Figure 2C), but lacking lymphoid or progenitor markers (Figure S4). Moreover, the cells displayed activation markers [54] including CD11a, CD11c, and MHC II (Figure 2C). Based on immunohistochemical analysis, we found that most of the GFP^+^ infiltrating cells were concentrated within the margins of the lesion (Figure 2D). These marginal infiltrating monocyte-derived MΦ acquired an activated morphology manifested by a large cell body with almost no processes, whereas the distal infiltrating cells demonstrated a multi-process morphology with a small cell body (Figure 2E). Notably, the infiltrating cells at the margins were Gr1^+^(Ly6C^+^), whereas the distal ones were Gr1^−^ (Ly6C^−^) (Figure 2F). Moreover, monocyte-derived MΦ at the margins of the lesion, unlike infiltrating cells (GFP^+^) distal to it, expressed CD11c (Figure 2G), a β-integrin expressed by activated MΦ at inflammatory lesions in the spinal cord [55]. Flow cytometric analysis of dissected spinal cord tissues confirmed the expression of CD11c by MΦ (CD11b^+^), including both infiltrating MΦ (CD45.1^+^) and activated resident microglia (CD45.1^−^), at the lesion area, but not distal to it (Figure 2H). However, the localization of the CD11c^+^ microglia changed as recovery progressed; at early time points, CD11c expressing microglia were confined to the margins (Figure 2G), whereas later (from d14 onward) these cells were also found at the epicenter of the lesion (Figure S5).

### Macrophages Derived from Infiltrating Monocytes Are Involved in the Process of Recovery from Spinal Cord Injury

**Figure 3:**
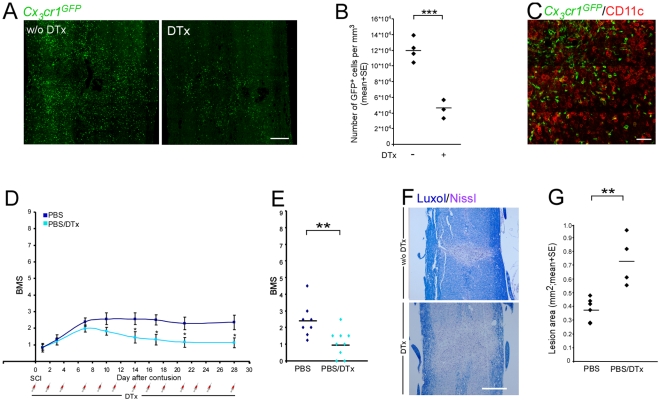
Infiltrating monocyte-derived macrophages are involved in the spontaneous process of functional recovery from spinal cord injury. [*CD11c*-DTR: *Cx_3_cr1* ^GFP/+^>wt] BM chimeras were generated by reconstitution of irradiated C57BL/6J mice with *CD11c*-DTR: *Cx_3_cr1* ^GFP/+^ BM. All mice were subjected to SCI, and half of them received IP injections of DTx starting immediately after the injury, then every other day. (A) GFP staining of the injury site to detect the presence of monocyte-derived MΦ, without (w/o; left panel) or with (right panel) DTx ablation (scale bar = 250 µm). (B) Quantitative analysis of monocyte-derived MΦ (number of GFP^+^ cells) at the injured site. Treatment with DTx significantly reduced the number of monocyte-derived MΦ (Student's *t*-test; *t* = −7.39; df = 5; *p* = 0.0007). (C) Representative micrograph of injured spinal cord sections, labeled for monocyte-derived MΦ by GFP (green) and for CD11c (red). The sections were taken from DTx-treated animals, showing that the remaining GFP^+^ cells following ablation are CD11c negative, whereas the remaining CD11c^+^ cells are GFP^−^, representing the resident microglia (scale bar = 100 µm). (D) Hind-limb locomotor performance was assessed according to the BMS (repeated measures ANOVA; F[between groups]_1,15_ = 4.88; *p* = 0.04). (E) Mean locomotor score (BMS) of individual mice on d28 after SCI (Student's *t*-test; *t* = −2.9; df = 5; *p* = 0.01). (F) Staining for myelin by Luxol (blue), and nuclei by Nissl (pink) both in the absence (w/o; upper panel) or in the presence (lower panel) of DTx (scale bar = 250 µm). (G) Quantitative analysis of the size of the injury site as a function of treatment with DTx, determined by Luxol and Nissl staining. Ablation of infiltrating CD11c^+^ monocyte-derived MΦ resulted in increased lesion size following SCI (Student's *t*-test; *t* = 3.6; df = 3.82; *p* = 0.02). *y*-Axis error bar represents SE. The assessment of the functional outcome is from one experiment, representative of two independent experiments performed.

We next wished to investigate the role of these infiltrating cells in functional recovery. Since the spontaneously recruited monocyte-derived MΦ, located at the margins of the lesion site, expressed CD11c, we implemented a conditional in vivo cell ablation strategy targeting cells by virtue of their *CD11c* promoter activity. Specifically, we generated [*CD11c*-DTR: *Cx_3_cr1* ^GFP/+^>wt] BM chimeras whose wt BM was replaced by BM of mice that carry both the GFP insertion in the *Cx_3_cr1* locus, and a DTR transgene under control of the *CD11c* promoter [39]. In the resulting mice, GFP expression could be used to trace the CNS infiltrates, and administration of DTx allowed the elimination of only the infiltrating monocyte-derived MΦ that expressed CD11c while sparing CD11c^+^ resident microglia. [*CD11c*-DTR: *Cx_3_cr1* ^GFP/+^>wt] BM chimeras were subjected to the SCI protocol 2 mo after BM transplantation. The DTx was given immediately following the injury every other day to ensure depletion of all BM-derived cells that could be potentially recruited to the injury site. We confirmed that DTx treatment resulted in specific depletion of infiltrating monocyte-derived cells (CD11c^+^) at the injured cord (Figure 3A, 3B), but spared the resident CD11c^+^ microglia (GFP^−^) (Figure 3C). To assess recovery in the absence of CD11c^+^ monocyte-derived MΦ, we performed a similar experiment in which half of the mice received serial IP injections of DTx during the entire recovery period. Although under our experimental conditions of severe SCI spontaneous recovery was very limited, treatment with DTx further diminished this limited recovery of hind limb motor function (Figure 3D, 3E). As a corollary, depletion of the monocyte-derived MΦ resulted in a greater spread of damage, manifested by larger lesion size as determined by Luxol-Nissl staining (Figure 3F, 3G). Taken together, the results presented in Figure 1, showing that increasing the peripheral pool of naïve monocytes enhanced recovery, with the impaired spontaneous recovery following depletion of CD11c-expressing MΦ derived from infiltrating peripheral monocytes, suggest that CD11c^+^ descendants of spontaneously infiltrating monocytes contribute to the recovery process following SCI. These findings raised the further question as to why the spontaneous recovery is limited: is it due to the suboptimal number, to timing, or to activity of the infiltrating monocyte-derived MΦ?

### Vaccination Augments Spontaneous Monocyte Infiltration to the Injured Spinal Cord

**Figure 4:**
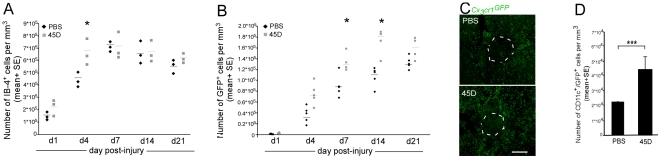
Vaccination results in enhanced infiltrat? ion of monocyte-derived macrophages to the injured spinal cord. [*Cx_3_cr1* ^GFP/+^>wt] BM chimeric mice were generated by reconstitution of irradiated C57BL/6J mice with bone marrow cells from *Cx_3_cr1* ^GFP/+^-transgenic mice. Half of these chimeras were vaccinated with myelin-derived altered peptide ligand (45D) 7 d before the SCI. Spinal cord sections were prepared from mice killed 1, 4, 7, 14, and 21 d after injury. (A) Quantitative analysis of cells labeled with IB-4, to identify both resident microglia and monocyte-derived MΦ, at different time points after the contusion, in either vaccinated (gray bar) or unvaccinated (black bar) mice (ANOVA; F_9,20_ = 16.7, *p* = 0.0001). (B) Kinetics of monocyte-derived MΦ infiltration to the injured site, measured by the number of GFP^+^ cells in both vaccinated (gray bar) and unvaccinated (black bar) mice. Vaccination significantly enhanced the monocyte-derived MΦ infiltration to the injured site (ANOVA; F_9,37_ = 52.12; *p* = 0.0001). (C) Sections from [*Cx_3_cr1* ^GFP/+^>wt] BM chimeras labeled for GFP (representing infiltrating monocyte-derived MΦ; green) in unvaccinated (upper panel) and vaccinated mice (lower panel) (scale bar 250 µm). (D) Quantitative analysis of GFP^+^/CD11c^+^ cells at the injury site revealing increased numbers of infiltrating CD11c^+^ monocyte-derived MΦ following vaccination (Student's *t*-test; *p*<0.001). In the kinetic analysis, asterisks indicate significant differences between vaccinated and unvaccinated groups. Significant differences were also found between different time points. *y*-Axis error bar represents SE. The dashed line in (C) that demarcates the lesion site was determined according to GFAP immunoreactivity.

To address the factors involved in the limited spontaneous recovery, we chose an experimental paradigm involving T cell–based vaccination with a myelin-derived peptide, which has been shown to significantly enhance recovery from SCI, while avoiding the induction of experimental autoimmune encephalomyelitis [46],[56],[57]; this vaccination procedure is associated with the activation of microglia/MΦ at the injured site [47],[48],[58],[59]. We hypothesized that this vaccination protocol (using MOG-derived altered peptide [35]–[55; MOG–45D]) might affect monocyte infiltration, and thus would allow us to test whether one of the limiting factors in the spontaneous recovery is related to the process of monocyte-derived MΦ recruitment. To that end, we analyzed infiltration of monocytes in spinally injured chimeric mice that were vaccinated 1 wk before the injury. The analysis of SCI lesions of [*Cx_3_cr1* ^GFP/+^>wt] BM chimeras revealed that the number of IB-4^+^ cells increased during the period of assessment in both the vaccinated and untreated mice (Figure 4A). Analysis for the presence of the GFP^+^ cellular infiltrate revealed that from d4 onward, there was a higher level of monocyte-derived MΦ in the immunized group compared to the control (Figure 4B, 4C). The effect of the vaccination on monocyte-derived MΦ recruitment was confirmed by flow cytometric analysis of [CD45.1>CD45.2] BM chimeras (1,698±361 versus 3,046±471 CD45.1^+^/CD11b^+^ infiltrating cells/mg tissue [mean±SE], in control and vaccinated groups respectively; Student's *t*-test *p*<0.05). Furthermore, the vaccination not only increased the total number of infiltrating monocyte-derived MΦ but also the abundance of these cells that expressed CD11c (Figure 4D). In accordance with the previously reported effects on recovery [47],[48], the enhanced monocyte-derived MΦ recruitment was specific for vaccination with a CNS-related antigen (45D), and was not seen following immunization with an irrelevant antigen (OVA), or challenge with CFA or PBS alone (Figure S6).

### Enhanced Recovery, Achieved by Vaccination, Is Dependent on Augmentation of Spontaneous Monocyte Infiltration to the Injured Spinal Cord

**Figure 5:**
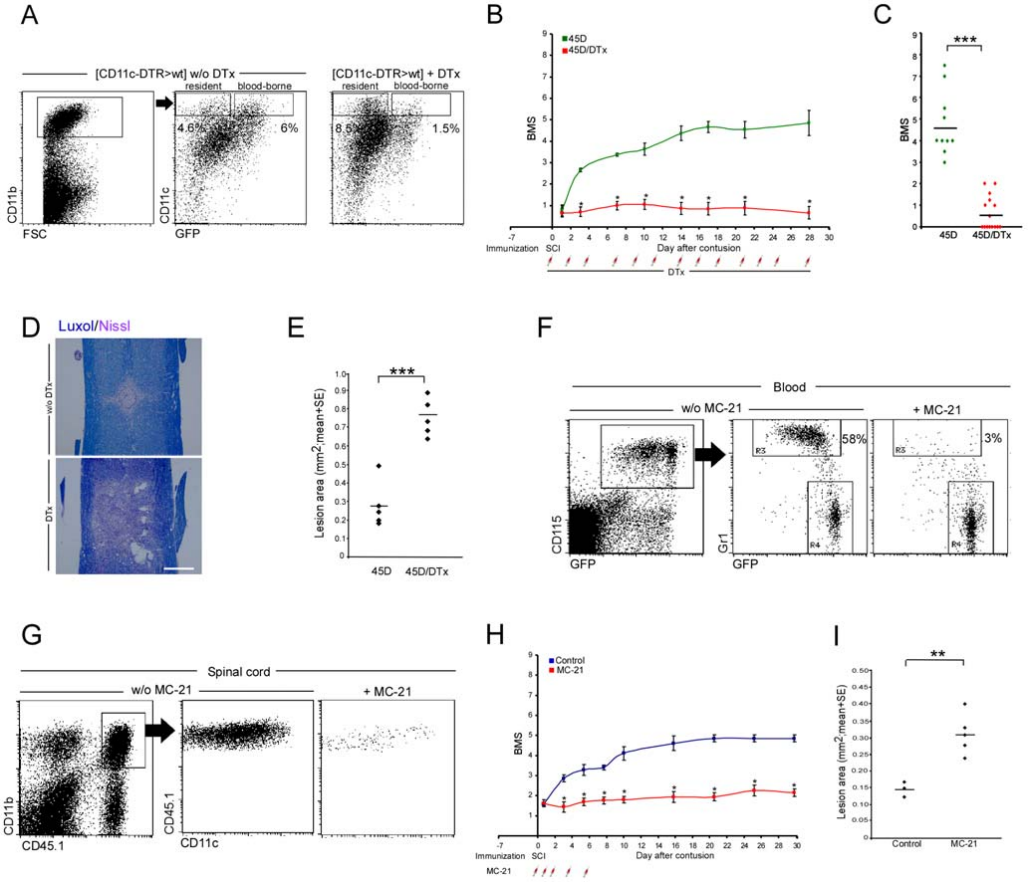
Conditional ablation of monocyte-derived macrophages at the injured site or antibody-mediated monocyte-depletion in the peripheral blood abolishes the augmented recovery in a paradigm of vaccination. (A–E) [*CD11c*-DTR: *Cx_3_cr1* ^GFP/+^>wt] BM chimeras were vaccinated 7 d before SCI and divided into two groups; one group was left untreated and one group was injected IP with DTx every other day starting immediately after the injury. (A) Flow cytometric analysis of lesion sites in injured DTx-treated and non-treated [*CD11c*-DTR>wt] BM chimeras. Note specific ablation of CD11c^+^ infiltrating monocyte-derived MΦ in DTx-treated mice, but persistence of CD11c^+^ resident microglia (GFP^−^). The cells in the dot plots to the right are gated according to CD11b expression, as indicated. (B) Hind-limb locomotor performance was assessed according to the BMS (repeated measures ANOVA; F[between groups]_1,23_ = 144; *p* = 0.0001). (C) Mean locomotor score (BMS) of individual mice on d28 after spinal cord injury (Student's *t*-test; *t* = 8.6; df = 12.21; *p*<0.0001). (D) Staining for myelin by Luxol (blue), and nuclei by Nissl (pink) in the absence (w/o; upper panel) or in the presence (lower panel) of DTx (scale bar = 250 µm). (E) Quantitative analysis of the size of the injury site as a function of treatment with DTx, determined by Luxol and Nissl staining. Ablation of infiltrating CD11c^+^ monocyte-derived MΦ resulted in significantly increased lesion size following SCI (Student's *t*-test; *t* = −6.5; df = 8; *p* = 0.0002). (F–I) Depletion of Ly6C(Gr1^+^)CCR2^+^ monocytes in the blood, using MC-21 antibody (F), resulted in decreased recruitment of infiltrating monocytes to the injured cord (G), worsening of recovery (H; repeated measures ANOVA; F[between groups]_1,11_ = 73.623; *p* = 0.0001), and larger lesion size compared to control group (I; Student's *t*-test; *t* = −3.77; df = 4.2; *p* = 0.017). Asterisks denote statistically significant differences between the indicated groups in (C), (E), and (I), and compared to the relevant controls in (B) and (H). *y*-Axis error bar represents SE. The assessment of the functional outcome presented is from one experiment representative of four independent experiments performed.

Having demonstrated that vaccination induced earlier and augmented infiltration to the injured spinal cord of monocyte-derived MΦ expressing CD11c, we next used this paradigm to test whether this earlier increase of infiltration could be responsible for the observed enhanced recovery. We therefore adopted again the conditional in vivo cell ablation strategy, targeting cells by virtue of *CD11c* promoter activity. We vaccinated [*CD11c*-DTR: *Cx_3_cr1* ^GFP/+^>wt] BM chimeric mice and 1 wk later subjected all mice to SCI. The animals were then separated into two groups (see Methods for details); one group received treatment with DTx along the entire recovery period and one group remained untreated. The efficiency of the ablation and its specificity for CD11c^+^/GFP^+^ monocyte-derived MΦ, but not activated CD11c^+^/GFP^−^ microglia, were confirmed by flow cytometry (Figure 5A; 6% versus 1.5% CD11c^+^/GFP^+^ cells out of CD11b^+^ cells without versus with DTx treatment, respectively) and histology (Figure S7A). The specific depletion of the CD11c^+^/GFP^+^ monocyte-derived MΦ resulted in increased numbers of CD11c^+^ resident microglia (Figure 5A; 4.6% versus 8.5% CD11c^+^/GFP^−^ cells out of CD11b^+^ cells without versus with DTx treatment, respectively), suggesting that the infiltrating monocyte-derived MΦ regulated activated resident microglia. We did not observe any significant effect on other inflammatory cell populations by the DTx treatment (Figure S7B, S7C). Importantly, in accordance with our previous studies, the vaccination resulted in enhanced recovery (Figure 3D, dark blue, versus Figure 5B, green), which was completely abrogated when the activated monocyte-derived CD11c^+^ MΦ were ablated (Figure 5B, 5C). To substantiate the functional BMS results, we analyzed the size of the lesions 21 days after injury by Luxol-Nissl staining. Spinal cords of vaccinated, DTx-treated mice showed significantly enlarged lesions as compared to injured chimeric controls (Figure 5D, 5E). Interestingly, impairment of recovery was evident earlier in the vaccinated mice than in animals that were allowed to recover spontaneously (Figure 5B versus Figure 3D), in correlation with the earlier monocyte infiltration and appearance of CD11c^+^/GFP^+^ cells in close proximity to the injury site (Figure 4B, 4D). Notably, the vaccinated mice showed improvement in motor function similar to that of the wt mice that were adoptively transferred with naïve monocytes (Figure 1D, 1E). To exclude a general toxic effect of the DTx that could directly cause a decline in motor function, we repeated the experiment using wt C57BL/6J mice that are resistant to DTx. Repeated injections of DTx starting immediately after SCI had no effect on functional motor recovery in these animals (Figure S8), supporting the reliability and specificity of this ablation procedure. The fact that both recovery and monocyte recruitment were augmented using the vaccination paradigm encouraged us to use this paradigm as a more sensitive system to further study the involvement of infiltrating monocytes in the recovery process. Therefore, MOG-45D vaccination was used in all further experiments.

To provide additional independent evidence for the critical role of infiltrating monocyte-derived MΦ in the recovery from SCI, we took advantage of the anti-CCR2 antibody MC-21, which selectively depletes from the peripheral blood the Ly6C^+^(Gr1^+^)CCR2^+^ monocyte subset that can potentially infiltrate to inflamed tissues [9],[50]. Wild-type mice were vaccinated with the 45D peptide 1 wk before the injury, and then half of the mice received the MC-21 treatment at d0, d1, d2, d4, and d6 postinjury. The treatment of 45D-immunized C57BL/6 wt mice with MC-21, which ablated their Ly6C^+^ monocytes in the circulation (Figure 5F), resulted in decreased monocyte recruitment to the lesion site (Figure 5G; 3,090±581 versus 293±108 CD45.1^+^/CD11b^+^ infiltrating monocyte-derived MΦ [mean±SE] without versus with MC-21 treatment, respectively; Student's *t*-test; *t* = 5.6; df = 5; *p* = 0.0026), without significantly affecting the infiltration of other inflammatory cell types (Figure S9). This treatment abrogated the beneficial effect of the vaccination, as it resulted in impaired motor function (Figure 5H) and increased lesion size (Figure 5I) compared to the control group. Collectively, the data from these two independent depletion experiments established the critical contribution of monocyte-derived CD11c-expressing MΦ in the recovery process following SCI.

**Figure 6:**
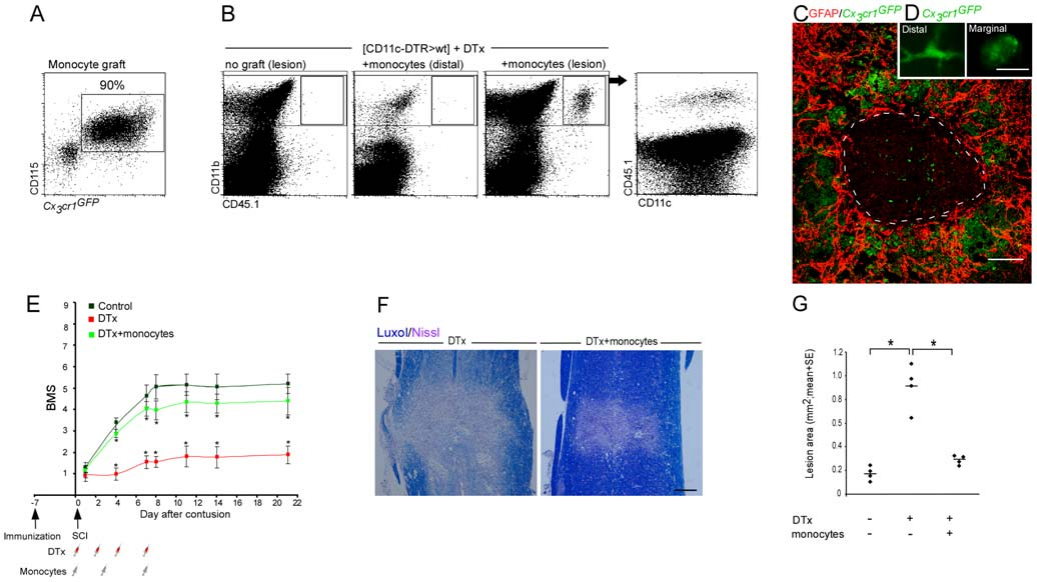
Replenishment of the monocyte pool restores functional recovery from spinal cord injury. Vaccinated [*CD11c*-DTR>wt] BM chimeras were subjected to SCI and subsequently divided into three groups; one group remained untreated and served as a control. The two others were treated with DTx during the first week after injury with or without replenishment of their monocyte pool with *Cx_3_cr1* ^GFP/+^ monocytes (CD45.1). (A) Flow cytometric analysis of the naïve monocyte graft, isolated from the BM, prior to its injection to the injured mice. Note that the vast majority of cells represent *Cx_3_cr1* ^GFP/+^/CD115^+^ monocytes. (B) Flow cytometric analysis of spinal cord samples to identify descendents of grafted monocytes (CD45.1^+^). Note specific homing of transferred monocytes to the lesion area. The dot plot to the right is gated on CD11b^+^ MΦ indicating CD11c expression on both grafted and endogenous cells. (C) Immunohistological analysis of engrafted cells (GFP^+^; green), demonstrating their preferential localization to the margins of the lesion site demarcated by GFAP (red). (D) Morphological differences between marginal (right) and distal (left) engrafted cells (GFP^+^; green). (E) Mean locomotor score (BMS) for each group as a function of time postinjury. Adoptive transfer of monocytes resistant to ablation administered in parallel to DTx treatment during the first week restored recovery (repeated measures ANOVA; F[between groups]_2,29_ = 15.97; *p* = 0.0001). (F) Staining for myelin by Luxol (blue), and nuclei by Nissl (pink) of sections taken from DTx-treated mice with or without adoptive transfer of monocytes. (G) Quantitative analysis of the size of the injury site as a function of monocyte transfer, determined by Luxol and Nissl staining. Adoptive transfer of monocytes significantly reduced the size of the lesion site compared to the group that was DTx-ablated and did not receive monocytes (ANOVA; F_2,9_ = 42.17, *p* = 0.0001). Asterisks indicate significant differences compared to the relevant controls in (E) and between the indicated groups in (G). *y*-Axis error bar represents SE. Scale bar representation: (C) 100 µm; (D) 10 µm; and (F) 200 µm. The dashed line that demarcates the lesion site was determined according to GFAP immunoreactivity. The assessment of the functional outcome presented is from one experiment representative of two independent experiments performed.

To provide further confirmation that the depletion of monocyte-derived MΦ was the reason for the impaired recovery following DTx administration, we examined whether restoration of the monocyte pool would reverse the functional loss that resulted from this depletion. We therefore tested whether complementation of the DTR transgenic monocytes with wt monocytes that do not harbor the *CD11c*-DTR transgene, and whose descendants would hence be resistant to the toxin treatment, could reverse the DTx-induced loss of recovery. Since we found that the depletion of monocyte-derived MΦ starting on the second week and onward did not impair recovery (Figure S10), we injected the [*CD11c*-DTR>wt] BM chimeras with DTx and transferred the resistant *Cx_3_cr1* ^GFP/+^ (CD45.1) monocytes (Figure 6A) in this experiment only during the time frame of the first week (DTx was injected on d0, d2, d4, and d7; monocytes were administrated on d0, d3, and d7 post injury). In this experiment, all animals were vaccinated 1 wk before the SCI and then divided into three groups; one group was left untreated and served as a control, and the two others were subjected to DTx-mediated ablation. One of the DTx-treated groups received a passive transfer of wt (DTx-resistant) monocytes. Flow cytometric analysis on d7 revealed the accumulation of graft-derived MΦ that expressed CD11c at the lesion area, but not distal to it (Figure 6B). Immunohistochemical analysis showed the localization of these cells at the lesion margins; similar to the endogenous infiltrating myeloid cells, the graft-derived GFP^+^ MΦ hardly penetrated the epicenter of the lesion site and acquired distinct morphologies depending on their location (Figure 6C, 6D). Follow-up of locomotion activity of animals that were similarly treated revealed that the adoptively transferred wt monocytes restored the lost functional recovery of DTx-treated [*CD11c*-DTR>wt] BM chimeras (Figure 6E). The BMS results correlated with histological data showing significantly smaller lesions in DTx-treated mice that had received monocytes, as compared to mice that received DTx but were not replenished with monocytes (Figure 6F, 6G). Of note, these results provide further evidence against a general toxic mechanism as a cause for the functional loss induced by the depletion procedure.

### Macrophages Derived from Infiltrating Monocytes Display Immunoregulatory Features

**Figure 7:**
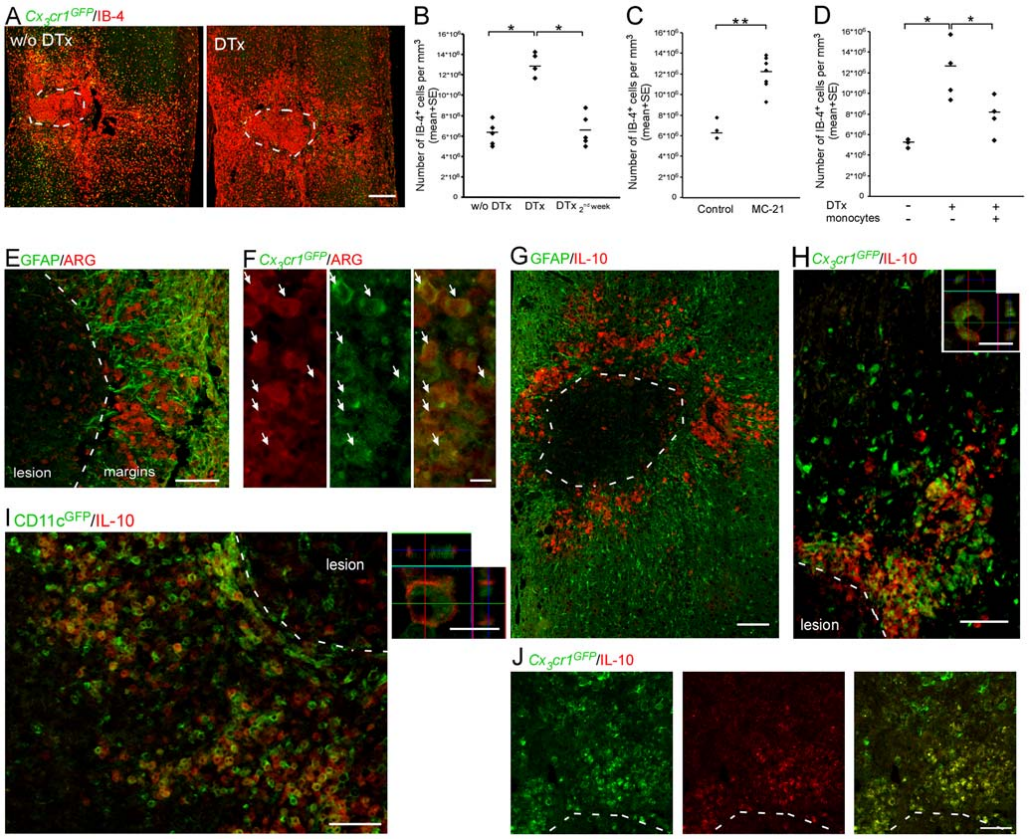
Infiltrating monocyte-derived macrophages exhibit an immunoregulatory phenotype. [*CD11c*-DTR: *Cx_3_cr1* ^GFP/+^>wt] BM chimeras were treated and analyzed as follows. (A) Labeling of spinal cord sections with IB-4 (red) to detect the presence of local inflammation, in DTx untreated (w/o), and treated mice. (B, C) Quantitation of IB-4^+^ cells representing local inflammation, in mice treated with DTx along the entire recovery process, only during the second week, or untreated (B; ANOVA; F_2,11_ = 35.69; *p* = 0.0001); or with and without treatment with MC-21 (C; Student's *t*-test; *t* = −4.9; df = 8; *p* = 0.001). (D) Quantification of the number of IB-4^+^ cells in injured mice, with and without adoptive transfer of DTx-resistant monocytes given in parallel with the DTx treatment (ANOVA; F_2,8_ = 6.1; *p* = 0.025). (E) Arginase I–expressing cells (ARG; red) are restricted to the margins of the lesion site demarcated by GFAP (green). (F) In [*Cx_3_cr1* ^GFP/+^>wt] BM chimeras, infiltrating monocyte-derived MΦ (GFP^+^; green) expressed arginase I (ARG; red), arrows indicate double-labeled cells. (G) Interleukin 10 (IL-10; red) expression is confined to the margins of the lesion site demarcated by GFAP (green). (H) In [*Cx_3_cr1* ^GFP/+^>wt] BM chimeras, monocyte-derived MΦ (GFP^+^; green) expressed IL-10 (red) only in close proximity to the lesion site. Right panel represents *z*-axis projection of individual cell. (I) In [*CD11c*-GFP>wt] BM chimeras, monocyte-derived MΦ that expressed CD11c (GFP^+^; green) coexpressed IL-10 (red). Right panel represents *z*-axis projection of individual cell. (J) Graft-derived MΦ (GFP^+^) expressing interleukin 10 (IL-10; red) at the margins of the lesion site following adoptive transfer to DTx-treated mice. Asterisks denote statistically significant differences between the indicated groups. *y*-Axis error bar represents SE. Scale bar in (A) represents 250 µm; in (E), (G), (H), and (I), 100 µm; in (J), 50 µm; and in (F) and right panels of (H) and (I), 10 µm. The dashed line that demarcates the lesion site was determined according to GFAP immunoreactivity.

To gain insight into the mechanism by which the infiltrating monocyte-derived MΦ, located at the margins of the lesion site, contribute to recovery, we examined whether and how their ablation affected the level of activated resident microglia. Interestingly, both the DTx-mediated ablation of monocyte-derived MΦ and the depletion of Ly6C^+^(Gr1^+^) monocytes from the peripheral blood caused an overall increase in the number of IB-4^+^ cells, indicating enhanced activation of the resident microglia in the absence of infiltrating monocyte-derived MΦ (Figure 7A–7C). Furthermore, transfer of wt monocytes following ablation restored the regulation of the local immune response, as the numbers of activated resident microglia were decreased to levels similar to those found in mice that were not treated with DTx (Figure 7D). These results suggested that the beneficial effect of the infiltrating monocyte-derived MΦ might be due to a regulatory role in controlling the local inflammation, induced following SCI.

To investigate the factors mediating this regulatory effect, we tested whether these cells exhibited characteristics associated with immune-regulating activities [60],[61]. Interestingly, analysis of lesion sites revealed that the monocyte-derived MΦ that were located at the margins of the lesion site, but not the distal ones, expressed arginase-I (Figure 7E, 7F) and the anti-inflammatory cytokine IL-10 (Figure 7G–7I). Moreover, ablation of CD11c-expressing monocyte-derived MΦ resulted in an overall decreased level of IL-10 at the injury site of [*CD11c*-DTR>wt] BM chimeras (Figure S11). Furthermore, adoptively transferred naïve monocytes that were injected to the DTx-treated mice, and were shown to restore recovery, also expressed similar immunoregulatory features upon encountering the lesion margins (Figure 7J).

### The Contribution of Infiltrating Monocyte-Derived Macrophages to the Recovery Process Is Crucially Dependent upon Their Expression of IL-10

**Figure 8:**
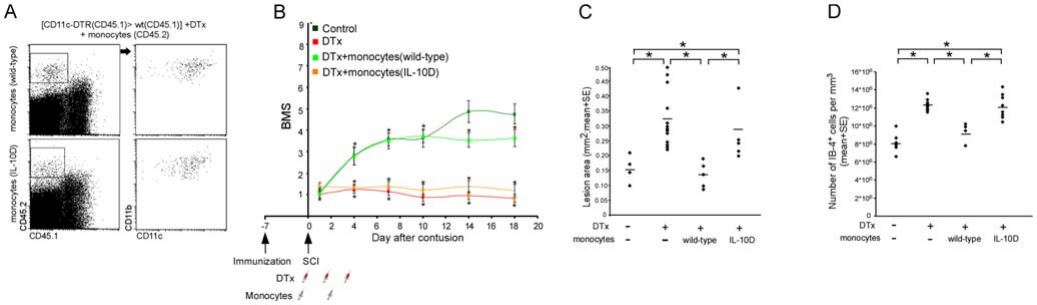
The contribution of monocyte-derived macrophages to the healing process is crucially dependent upon their expression of IL-10. [*CD11c*-DTR (CD45.1)>CD45.1] BM mice were vaccinated with 45D, a week later subjected to SCI, and then divided into four groups. One group remained untreated and served as a control. The three other groups were treated during the first week, with DTx and with or without administration of DTx-resistant monocytes isolated from either wt or IL-10–deficient mice. (A) Flow cytometric analysis of engrafted wt or IL-10– deficient (IL-10D) monocytes (CD45.2) found at the lesion area of vaccinated [*CD11c*-DTR (CD45.1)>CD45.1] BM chimeras treated with DTx. The cells in the dot plots to the right were gated according to CD45.2 expression, as indicated. MΦ derived from wt and IL-10–deficient monocytes were located at the lesion area, and expressed CD11c to a similar extent (284.4±45.9 versus 301.4±10.8 CD11b^+^/CD45.2^+^ [mean±SE] engrafted IL-10 deficient and wt MΦ, respectively; Student's *t*-test; *t* = 0.36; df = 4.4; *p* = 0.73). (B) Mean locomotor score (BMS) for each group as a function of time post injury (repeated measures ANOVA; F[between groups]_3,33_ = 23.4; *p* = 0.0001). (C) Quantitative analysis of the size of the injury site, determined according to Luxol-Nissl staining, as a function of the IL-10 expression by the transferred monocytes (wt; IL-10D) (ANOVA; F_3,29_ = 8.32, *p* = 0.0004). (D) Quantification of the number of IB-4^+^ cells in the lesion area of the different groups (ANOVA; F_3,24_ = 21.05; *p* = 0.0001). Asterisks denote statistically significant differences between the indicated groups in (C) and (D), and compared to the relevant controls in (B). *y*-Axis error bar represents SE. The assessment of the functional outcome presented is from one experiment representative of two independent experiments performed.

To investigate the specific contribution of IL-10 to the recovery-promoting activity of monocyte-derived MΦ, we compared the functional recovery of DTx-treated mice that were engrafted with wt monocytes to that of mice that were engrafted with monocytes derived from IL-10–deficient mice. [*CD11c*-DTR>wt] BM chimeric mice were immunized with 45D and subjected a week later to SCI. After the injury, the mice were separated into four groups: one group was left untreated, one group was treated with DTx, and the other two groups received DTx and a passive transfer of DTx-resistant monocytes either from wt or from IL-10 deficient mice. The analysis of DTx-treated [*CD11c*-DTR (CD45.1)>wt (CD45.1)] BM chimeras that had received an adoptive transfer of CD45.2^+^ monocytes (IL-10 deficient or wt) established that graft-derived IL-10–deficient MΦ arrived at the lesion area and expressed CD11c, similarly to their wt counterparts (Figure 8A). However, in contrast to the engraftment with wt monocytes, transfer of IL-10-deficient monocytes failed to restore recovery, and resulted in similar motor function as that observed in the DTx-treated mice that did not receive monocytes (Figure 8B). Moreover, replenishment of IL-10–deficient monocytes did not reduce the lesion size nor restrict the activation of resident microglia (Figure 8C, 8D). Furthermore, the absence of IL-10 production by monocytes had no significant effect on the composition of other inflammatory infiltrates (Figure S12). These results establish that expression of IL-10 by monocyte-derived MΦ is a critical factor determining their ability to control the local immune response and to contribute to the motor function recovery.

## Discussion

In this study, we demonstrated that a population of MΦ derived from infiltrating monocytes located at the margins of the lesion site contribute to recovery following SCI by mediating an immunoregulatory role via the anti-inflammatory cytokine IL-10. These cells are pivotal and nonredundant players in the spontaneous recovery process following injury, as their CNS counterpart, the resident microglia, could not replace their functions. The physiological recruitment of the monocyte-derived MΦ to the injured spinal cord and their essential role in the recovery were demonstrated using a variety of independent techniques. We found that monocytes spontaneously infiltrate to the damaged CNS, and that their descendents preferentially accumulate at the margins of the lesion site. Augmenting the naïve monocyte pool by either adoptive transfer or CNS-specific vaccination resulted in improved recovery. By using antibody-mediated monocyte depletion in wt mice and conditional ablation of monocyte-derived MΦ in BM chimeras (resulting in the depletion of either naïve monocytes in the peripheral blood or their infiltrating descendents at the lesion margins, respectively), in a manner that spared the resident microglia, we demonstrated that monocyte-derived MΦ are pivotal for recovery. Reconstitution of the monocyte pool by wt monocyte transfer restored the improvement of motor function, whereas transfer of IL-10-deficient monocytes failed to do so.

Two recent publications have questioned the use of BM chimeras in CNS studies [8],[9]; irradiation, even in the absence of further injury, mobilized monocytes to the noninjured CNS. Our results are in line with these reports, though the numbers of these recruited cells were negligible relative to their massive infiltration following spinal cord injury. Nevertheless, the chimeras used throughout this study were prepared while shielding their heads during irradiation, a procedure that prevents any nonspecific infiltration of monocytes and therefore provides a reliable model for investigating monocyte recruitment following SCI. To unequivocally prove that this infiltration of monocytes resulted from the injury and not from the irradiation, we used passive transfer of naïve monocytes to nonchimeric wt mice, and demonstrated, in agreement with other studies [26],[62]–[64], the spontaneous infiltration of monocytes under CNS pathological conditions.

Immune cells, and in particular MΦ/monocytes, have been recognized as a heterogeneous population in terms of their functional role, beyond host defense, in the healing of peripheral organs. However, in the CNS, which is considered an immune-privileged site, the low levels of spontaneous recovery have been attributed, at least in part, to a robust and detrimental local inflammatory response [1],[6],[7],[10].

Our group demonstrated almost a decade ago that alternatively activated MΦ promote CNS recovery from injury; specifically, we showed that peripheral MΦ activated ex vivo by exposure to peripheral nerve tissue, when injected at a specific time frame after the injury to the margins of the lesion site, benefit repair [22],[65],[66]. Our finding that such MΦ are needed for repair was unexpected, primarily due to the fact that the site of injury is already laden with microglia/MΦ, and it was not clear what further functionality could be provided by the additional cells that were introduced. At that early stage of our research, it was not clear whether beneficial MΦ could be induced only by local administration of autologous ex vivo–activated MΦ, or alternatively, whether protective MΦ are spontaneously recruited as part of the endogenous repair mechanism, but in numbers that are insufficient within the critical therapeutic time frame.

The present study highlights the fact that infiltrating monocyte-derived MΦ and resident microglia differ in their distribution and activities following SCI. The resident activated microglia were distributed throughout the epicenter of the lesion and at its margins. In contrast, monocyte-derived MΦ that contributed to the repair process were largely excluded from the lesion center and preferentially located at its margins. Selective ablation of monocyte-derived MΦ resulted in increased numbers of resident activated microglia and impaired recovery. Thus, the increased number of microglia failed to compensate for the loss of infiltrating monocyte-derived MΦ, suggesting that these recruited cells have a unique role in the recovery process that cannot be replaced by the resident microglia. The distinctive roles of activated microglia and this subset of monocyte-derived MΦ may be related to the activation state of these myeloid cells prior to encountering the damaged tissue; the resident microglia are embedded in the CNS prior to the injury and are immediately activated by the insult, whereas naïve infiltrating monocyte-derived MΦ do not encounter the injured CNS tissue prior to their delayed arrival to the damage site [67]. Notably, although we followed the recruitment of naïve monocytes, our present study is consistent with the previous report of Rapalino et al. [22], which reported that preactivation of the transplanted MΦ is crucial for their beneficial effect; we demonstrate here that in order to be supportive, the infiltrating monocyte-derived MΦ are locally activated to acquire a nonclassical phenotype, probably en route to the site or within the local microenvironment of the lesion. Yet, the present study identifies the beneficial MΦ as part of the endogenous response to the insult, and also shows that these nonclassically activated MΦ serve an anti-inflammatory role.

Our present results suggest that the recovery following spinal injury involves monocyte-derived MΦ, yet these cells, at their spontaneous levels and activation state, are not sufficient. Increasing the naïve-monocyte pool by either adoptive transfer or CNS-specific vaccination resulted in a higher number of spontaneously recruited cells and improved recovery. This suggests that at least one of the limiting factors in the beneficial involvement of innate immune cells following CNS injury is the availability and/or the extent of the spontaneous recruitment of monocytes from the circulation. Importantly, although our current study identifies monocytes as key players in the recovery process, our findings do not contradict the established contribution of other immune components [11],[13],[14],[68]. Furthermore, the use of T cell–based vaccination allowed us to link monocyte involvement in the recovery process, identified in the current study, with the beneficial contribution of adaptive immunity that was previously established by our group [3],[69],[70]. However, it remains to be shown how the stimulation of the CNS antigen-reactive T cells in the vaccination protocol used, contributes to the enhanced monocyte recruitment, be it via local modifications at the lesion site or through systemic effects.

Previous studies, which reported enhanced recovery following depletion of MΦ, mediated by injection of liposome-encapsulated clodronate [1] or by blockage of their recruitment using anti-integrin antibodies [6],[7],[10],[71]–[73] or chemokine antagonists [74], suggested that myeloid cells are detrimental to tissue recovery. The techniques used in those studies resulted in nonselective depletion or prevented recruitment of all MΦ/myeloid cells, regardless of their phenotype, activation, location, and, most importantly, origin. However, in the present study, we ablated only activated CD11c expressing monocyte-derived MΦ, but spared the resident microglia.

Interestingly, the monocytic infiltrate, whether spontaneous or enhanced by vaccination, was not detected immediately after injury. Moreover, ablation of monocyte-derived MΦ from the second week onward had no effect on functional recovery. Taken together, these two observations suggest that the essential effect of these cells is restricted to the first week following injury, and probably between d4 and d7 postinjury. Thus, our results do not refute the potential deleterious effect of other blood-derived cells or even of other subsets of infiltrating monocytes following SCI, but rather establish that assuming all MΦ population to be destructive at all time points is an inaccurate generalization.

Importantly, our work does not contradict other studies demonstrating the benefit attained by the restriction of local inflammation at a certain time point and by a specific subpopulation of immune cells [6],[7],[10],[75]–[77]. Our results, however, attribute a novel anti-inflammatory function to CD11c^+^ monocyte-derived MΦ, which benefit the injured CNS by controlling the local immune response, rather than harm it by adding to the already-detrimental inflammation. Such regulation depends on timing and location. Thus, our study further substantiates the notion that while the global suppression of the immune system denies its potential benefit, a controlled immune response is pivotal for preventing the spread of damage following CNS insult.

Histological examination of the SCI lesion sites showed that the monocyte-derived MΦ located at the margins of the lesion site expressed immunoregulatory factors, such as arginase-I and IL-10. Notably, they share these features with the so-called myeloid-derived suppressor cells (MDSC) [60],[78]–[81], which were originally identified in the context of tumor immune escape mechanisms [78]–[85], and more recently it has been suggested that they have a role in non-CNS tissue repair [36],[37] and in the resolution of autoimmune disease of the CNS [34],[86]. Importantly, our adoptive transfer experiments showed that IL-10-deficient monocyte-derived MΦ failed to promote recovery. This established that the anti-inflammatory cytokine IL-10, which dictates MDSC activities outside the CNS [79],[87], is a critical factor determining the beneficial function of the monocyte-derived MΦ in CNS recovery. Further studies are needed to establish whether these infiltrating monocyte-derived MΦ, identified here as essential for CNS recovery, are related to the MDSC population. Interestingly, ablation of the monocyte-derived MΦ seemed to affect the resident microglia. Thus, the SCI lesions of DTx-treated mice displayed a consistently significant increase in IB-4^+^ cells in the histological examinations. Moreover, following this ablation, flow cytometric analysis showed a higher percentage of activated CD11c^+^ resident microglia at the lesion area. Although further experiments are required to explore this crosstalk between the MΦ infiltrate and the resident microglia, this study argues against the general assumption that any increased infiltration of immune cells to the CNS will necessarily lead to enhanced destructive inflammation, but rather reveals that the infiltrating CD11c^+^ MΦ locally exhibit a phenotype that mediates the down-regulation of the local immune response. It is possible that other beneficial properties of infiltrating MΦ contribute to the repair process; MΦ are capable of secreting growth and neurotrophic factors [30]–[32],[41], phagocytosis and clearance of cell debris [88], degradation of growth-limiting scar tissue [89], and promotion of remyelination [32],[33],[90], all of which may lead to regeneration which is the most efficient repair process in the CNS.

Altogether, our study brings a new insight into the long-standing debate regarding the contribution of MΦ to CNS recovery. We defined a critical nonredundant role of a unique subset of infiltrating monocyte-derived MΦ that, at a specific location and time frame, mediate their beneficial activity through secretion of the immunoregulatory cytokine IL-10. This new understanding of the differential role of activated resident microglia and infiltrating monocyte-derived MΦ and their mutual relationship following CNS insult might enable the development of novel approaches to manipulate and refine the endogenous repair mechanisms, and thereby improve currently available therapies for CNS injuries.

## Supporting Information

Figure S1 **Infiltration of monocytes in head shielded chimeric mice results from the injury and not from the irradiation.** (A, B) Representative images showing GFP expression (A) and its quantification (B) in noninjured spinal cord of [*Cx_3_cr1* ^GFP/+^>wt] BM chimeric mice created with or without (w/o) head shielding (Scale bar: 250 µm). Results were verified using flow-cytometric analysis in [CD45.1>wt (CD45.2)] BM chimeras (132±21 versus 8±7 CD11b^+^ [CD45.1] infiltrating monocyte-derived MΦ per mg tissue [mean±SE], without and with head shielding, respectively; Student's *t*-test; *t* = 6.19; df = 4; *p* = 0.0035). The GFP or CD45.1 label allowed the identification of the BM-derived cells and their descendents in the congenic GFP^−^ or CD45.2 recipients. Head protection reduced the massive infiltration of myeloid cells to noninjured spinal cords (n.d; none detected). (C) Representative images of infiltrating myeloid cells (GFP^+^) in noninjured and injured spinal cord of [*Cx_3_cr1* ^GFP/+^>wt] BM chimeric mice prepared with head shielding (Scale bar: 250 µm). (D) Monocyte-derived MΦ (GFP^+^) were found along the central canal throughout its entire length (Scale bar: 50 µm). (E) Chimeric [wt>wt] and nonchimeric wt mice were subjected to SCI and adoptively transferred with CD45.1^+^ monocytes (CD115^+^). Quantification of CD11b^+^/CD45.1^+^ graft-derived cells based on flow cytometric analysis revealed that the same numbers of engrafted monocytes infiltrated to the injured spinal cord in nonchimeric and chimeric mice (Student's *t*-test; *t* = 0.21; df = 6; *p* = 0.84). *y*-Axis error bar represents SE.
(0.82 MB TIF)

Figure S2 **Confirmation of the efficiency of diphtheria toxin treatment by assessing the spleen.** The efficiency of DTx treatment is routinely verified in the periphery by assessing depletion of CD11c^+^/GFP^+^ cells in the spleen of [*CD11c*-DTR: *Cx_3_cr1* ^GFP/+^>wt] BM chimeras, in addition to their assessment in the injured cord.
(0.10 MB TIF)

Figure S3 **Monocyte-derived macrophage localization to the margins of the lesion is independent of the lesion severity.** Immunohistochemical analysis from [*Cx_3_cr1* ^GFP/+^>wt] BM chimeric mice that were subjected to a moderate contusive injury of the spinal cord (70 kdynes), showing the restricted accumulation of the infiltrating monocyte-derived MΦ (*Cx_3_cr1* ^GFP/+^; green) at the margins but not at the epicenter of the lesion site, similar to the distribution of these cells following the severe injury employed throughout this study. Scale bar = 100 µm.
(0.15 MB TIF)

Figure S4 **Monocyte-derived myeloid cells do not express lymphoid or progenitor markers.** Representative flow cytometric plots of various lymphoid/ progenitor markers by the myeloid infiltrating cells (CD11b^+^/ *Cx_3_cr1* ^GFP/+^/CD45.1^+^) in injured [*Cx_3_cr1* ^GFP/+^ (CD45.1)>wt (CD45.2)] BM chimeras (isotype control, gray line). The percentage of the positive cells within the infiltrating myeloid population is indicated above each marker.
(0.08 MB TIF)

Figure S5 **Expression of CD11c in the epicenter of the lesion site from d14 postinjury and onward.** Longitudinal sections labeled for IB-4 (red), GFP (green) and CD11c (yellow), showing that, at later time points (d14 and onward), CD11c expression by the resident microglia (IB-4^+^/GFP^−^ cells) was also observed at the epicenter of the lesion and not only at its margins. Right panel shows split images of the left panel. The dashed line demarcating the lesion site was determined according to GFAP immunoreactivity. Scale bar = 250 µm.
(1.49 MB TIF)

Figure S6 **Vaccination with a CNS-specific antigen, rather than an irrelevant antigen, is required to augment infiltration of monocyte-derived macrophages to the injured spinal cord.** [CD45.1>wt (CD45.2)] BM chimeras were vaccinated 7 d prior to spinal cord injury with: CNS specific antigen (45D/CFA), the irrelevant antigen ovalbumin (OVA/CFA), CFA, or PBS alone. The injured spinal cords were analyzed 1 wk after injury for the entrance of the infiltrating monocyte-derived MΦ (CD45.1^+^/CD11b^+^). Increased infiltration could be seen only in the mice that were vaccinated with CNS-specific antigen (ANOVA; F_3,9_ = 4.52; *p* = 0.03). Asterisk indicates significant differences between the indicated group to all other groups. *y*-Axis error bar represents SE.
(0.02 MB TIF)

Figure S7 **Diphtheria toxin depletes CD11c expressing monocyte-derived macrophages at the lesion area without significantly affecting infiltrates of other inflammatory cell types.** Analysis at the injured site 14 d postinjury of [*CD11c*-DTR: *Cx_3_cr1* ^GFP/+^>wt] BM chimeras, treated without (w/o) or with DTx ablation. (A) Assessment of monocyte-derived MΦ; GFP^+^ cells, ANOVA; F_3,6_ = 21.5; *p* = 0.0013), and (B, C) of infiltrates of other inflammatory cell types. Neutrophils were identified by gating on the CD11b^+^/Gr1^high^ and CD115^−^/GFP^−^ population, while T cells were assessed by gating on TCRβ^+^ cells (Student's *t*-test; *t* = −0.5; df = 5; *p* = 0.65 and *t* = −0.04; df = 3; *p* = 0.97, respectively). *y*-Axis error bar represents SE.
(0.12 MB TIF)

Figure S8 **DTx treatment of wt mice that do not harbor the** ***DTR*** **transgene does not inhibit functional recovery from spinal cord injury.** C57BL/6J mice were subjected to contusive SCI. Half of the animals were treated with DTx starting immediately after the injury. Locomotion was recorded at different time points following the injury, and is presented as the mean locomotor score (BMS) for each group. DTx administration to the nonchimeric (C57BL/6J) mice did not affect functional recovery following SCI (repeated measures ANOVA; F_1,18_ = 0.12; *p* = 0.73). *y*-Axis error bar represents SE.
(0.03 MB TIF)

Figure S9 **MC-21 treatment has no significant effect on other inflammatory cell types.** Quantitative analysis 14 d postinjury of neutrophils and T cells at the injured site of [*Cx_3_cr1* ^GFP/+^>wt] BM chimeras, without (w/o) or with MC-21 treatment. Neutrophils were identified by gating on CD11b^+^/Gr1^high^ and CD115^−^/GFP^−^ population, while T cells were assessed by gating on TCRβ+ cells (Student's *t*-test; *t* = −0.85; df = 5; *p* = 0.43 and *t* = 0.5; df = 5; *p* = 0.63, respectively). *y*-Axis error bar represents SE.
(0.02 MB TIF)

Figure S10 **Ablation of monocyte-derived macrophages in the second week or at the chronic phase following spinal cord injury does not affect recovery.** (A, B) [*CD11c*-DTR: *Cx_3_cr1* ^GFP/+^>wt] BM chimeras were vaccinated with 45D 7 d before SCI, and were treated with DTx during the second (2^nd^) week, along the entire period of recovery, or remained without DTx treatment. (A) Mean locomotor score (BMS) for each group as a function of time postinjury, showing that infiltrating monocyte-derived MΦ ablation does not affect recovery when it is carried out during the second week (repeated measures ANOVA; F[between groups]_2,26_ = 16.22; *p* = 0.0001). (B) Quantitative analysis of the size of the injury site as a function of treatment with DTx (ANOVA; F_2,10_ = 16.34; *p* = 0.007). (C) After verifying that monocyte-derived MΦ (GFP^+^) expressing CD11c were still found at the injury site 1 mo after injury, DTx was administered to immunized and nonimmunized mice DTx by repeated injections starting from 1 mo postinjury, when the animals had already reached plateau levels of recovery. The ablation of infiltrating monocyte-derived MΦ by DTx application at this late chronic stage had no effect on locomotor ability. Asterisks denote statistically significant differences between the indicated groups in (B) and compared to the relevant controls in (A). *y*-Axis error bar represents SE.
(0.11 MB TIF)

Figure S11 **Ablation of monocyte-derived macrophages results in reduction of IL-10 levels in the injured site.** (A, B) The levels of IL-10 at the injury site of [*CD11c*-DTR: *Cx_3_cr1* ^GFP/+^>wt] BM chimeras that were treated without (w/o) or with DTx were tested. (A) Representative micrographs of spinal cord sections labeled for IL-10, in the absence or presence of DTx treatment (scale bar = 100 µm). (B) Quantification of IL-10^+^ cells in the spinal cords with and without DTx treatment, based on immunohistochemical analysis (Student's *t*-test; *t* = −5.33; df = 6; *p* = 0.0018). *y*-Axis error bar represents SE.
(0.34 MB TIF)

Figure S12 **IL-10 deficiency in the infiltrating monocytes has no significant effect on other inflammatory infiltrates.** Quantitative analysis 14 d postinjury of neutrophils and T cells at the injury site of [*CD11c*-DTR:*Cx_3_cr1* ^GFP/+^>wt] BM chimeras treated with DTx in parallel to adoptive transfer of either wt or IL-10–deficient monocytes. Neutrophils were identified by gating on the CD11b^+^/Gr1^high^ and CD115^−^/GFP^−^ population, while T cells were assessed by gating on TCRβ^+^ cells (Student's *t*-test; *t* = 2.2; df = 4; *p* = 0.11 and *t* = −1.0; df = 6; *p* = 0.35, respectively). *y*-Axis error bar represents SE.
(0.03 MB TIF)

## Abbreviations

BMS: Basso Mouse Scale
BM: bone marrow
CFA: complete Freund's adjuvant
CNS: central nervous system
DTx: diphtheria toxin
DTR: diphtheria toxin receptor
GFAP: glial fibrillary acidic protein
IB-4: isolectin B4
MΦ: macrophage(s)
IL-10: interleukin 10
IP: intraperitoneal(ly)
IV: intravenous(ly)
MDSC: myeloid-derived suppressor cell
OVA: ovalbumin
SCI: spinal cord injury
SE: standard error
