# Supplementary figures and images for: Infiltrating Blood-Derived Macrophages Are Vital Cells Playing an Anti-inflammatory Role in Recovery from Spinal Cord Injury in Mice
Source: PLoS Med. 2009 Jul 28;6(7):e1000113. doi: 10.1371/journal.pmed.1000113 (PMC2707628; doi:10.1371/journal.pmed.1000113)

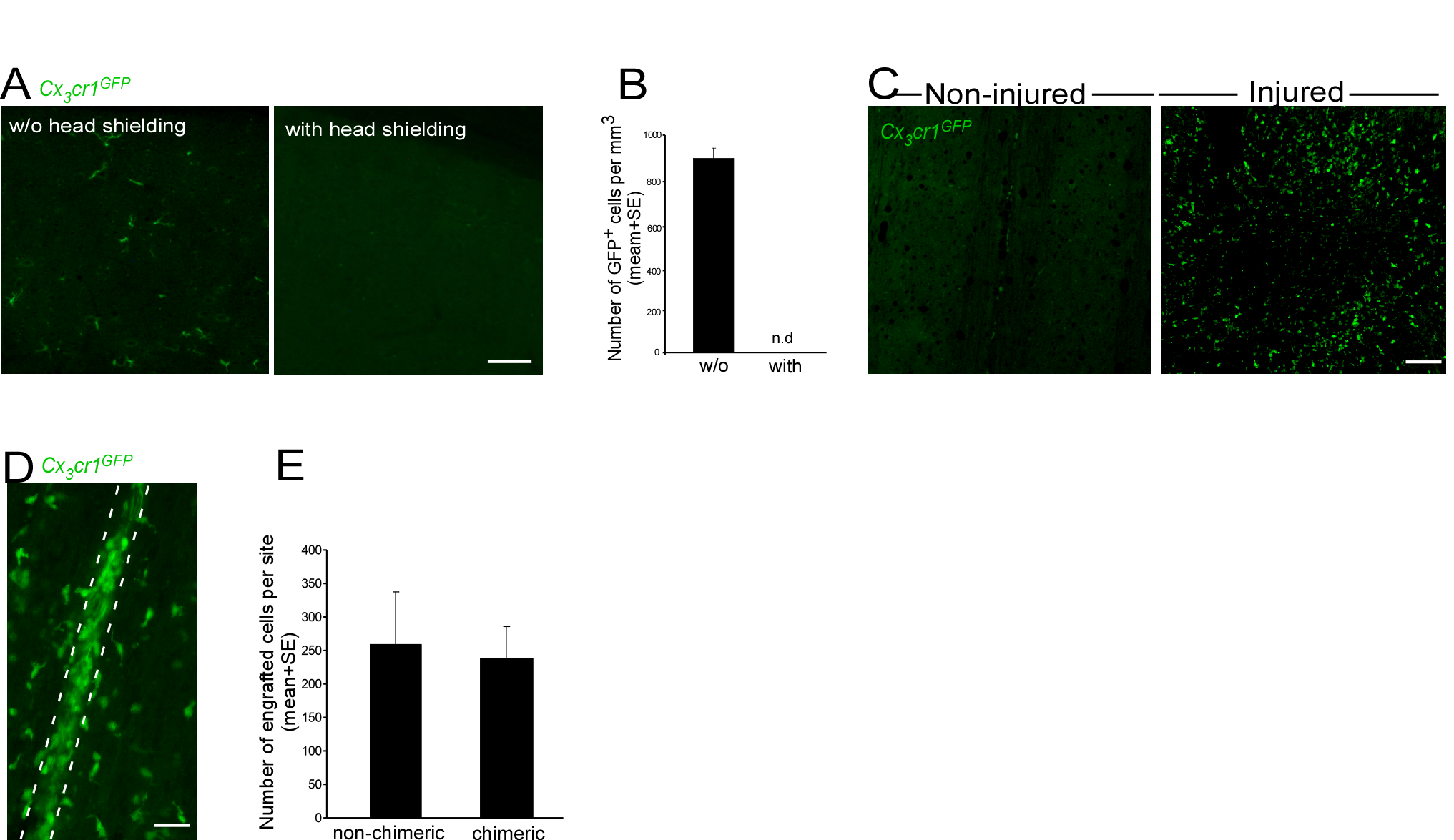

Supplement: Figure S1 — Infiltration of monocytes in head shielded chimeric mice results from the injury and not from the irradiation. (A, B) Representative images showing GFP expression (A) and its quantification (B) in noninjured spinal cord of [Cx3cr1 GFP/+>wt] BM chimeric mice created with or without (w/o) head shielding (Scale bar: 250 µm). Results were verified using flow-cytometric analysis in [CD45.1>wt (CD45.2)] BM chimeras (132±21 versus 8±7 CD11b+ [CD45.1] infiltrating monocyte-derived MΦ per mg tissue [mean±SE], without and with head shielding, respectively; Student's t-test; t = 6.19; df = 4; p = 0.0035). The GFP or CD45.1 label allowed the identification of the BM-derived cells and their descendents in the congenic GFP− or CD45.2 recipients. Head protection reduced the massive infiltration of myeloid cells to noninjured spinal cords (n.d; none detected). (C) Representative images of infiltrating myeloid cells (GFP+) in noninjured and injured spinal cord of [Cx3cr1 GFP/+>wt] BM chimeric mice prepared with head shielding (Scale bar: 250 µm). (D) Monocyte-derived MΦ (GFP+) were found along the central canal throughout its entire length (Scale bar: 50 µm). (E) Chimeric [wt>wt] and nonchimeric wt mice were subjected to SCI and adoptively transferred with CD45.1+ monocytes (CD115+). Quantification of CD11b+/CD45.1+ graft-derived cells based on flow cytometric analysis revealed that the same numbers of engrafted monocytes infiltrated to the injured spinal cord in nonchimeric and chimeric mice (Student's t-test; t = 0.21; df = 6; p = 0.84). y-Axis error bar represents SE. (0.82 MB TIF) [file pmed.1000113.s001.tif]

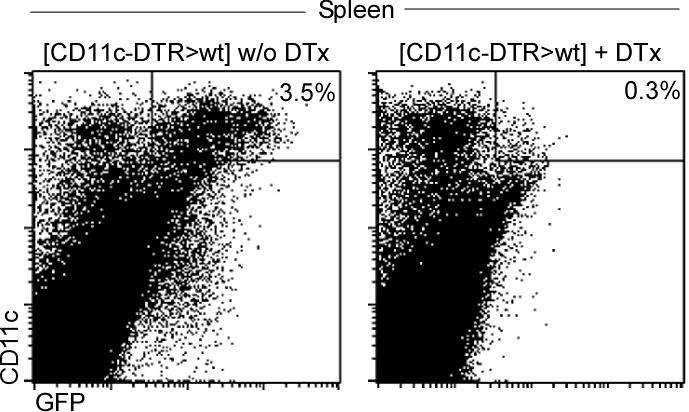

Supplement: Figure S2 — Confirmation of the efficiency of diphtheria toxin treatment by assessing the spleen. The efficiency of DTx treatment is routinely verified in the periphery by assessing depletion of CD11c+/GFP+ cells in the spleen of [CD11c-DTR: Cx3cr1 GFP/+>wt] BM chimeras, in addition to their assessment in the injured cord. (0.10 MB TIF) [file pmed.1000113.s002.tif]

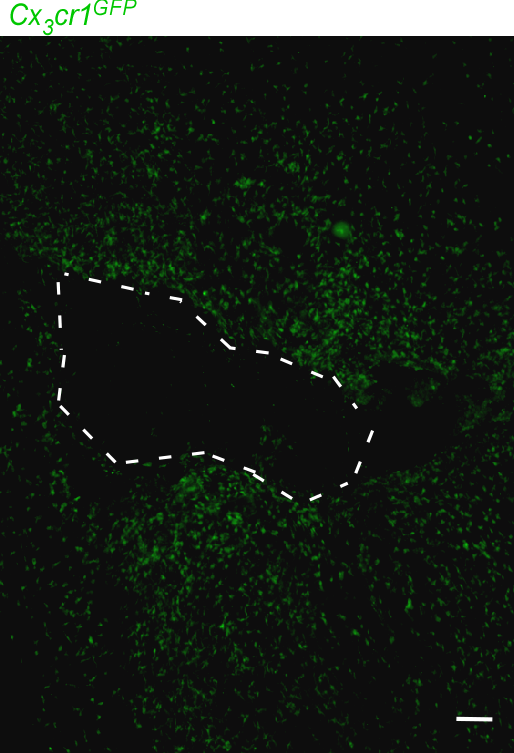

Supplement: Figure S3 — Monocyte-derived macrophage localization to the margins of the lesion is independent of the lesion severity. Immunohistochemical analysis from [Cx3cr1 GFP/+>wt] BM chimeric mice that were subjected to a moderate contusive injury of the spinal cord (70 kdynes), showing the restricted accumulation of the infiltrating monocyte-derived MΦ (Cx3cr1 GFP/+; green) at the margins but not at the epicenter of the lesion site, similar to the distribution of these cells following the severe injury employed throughout this study. Scale bar = 100 µm. (0.15 MB TIF) [file pmed.1000113.s003.tif]

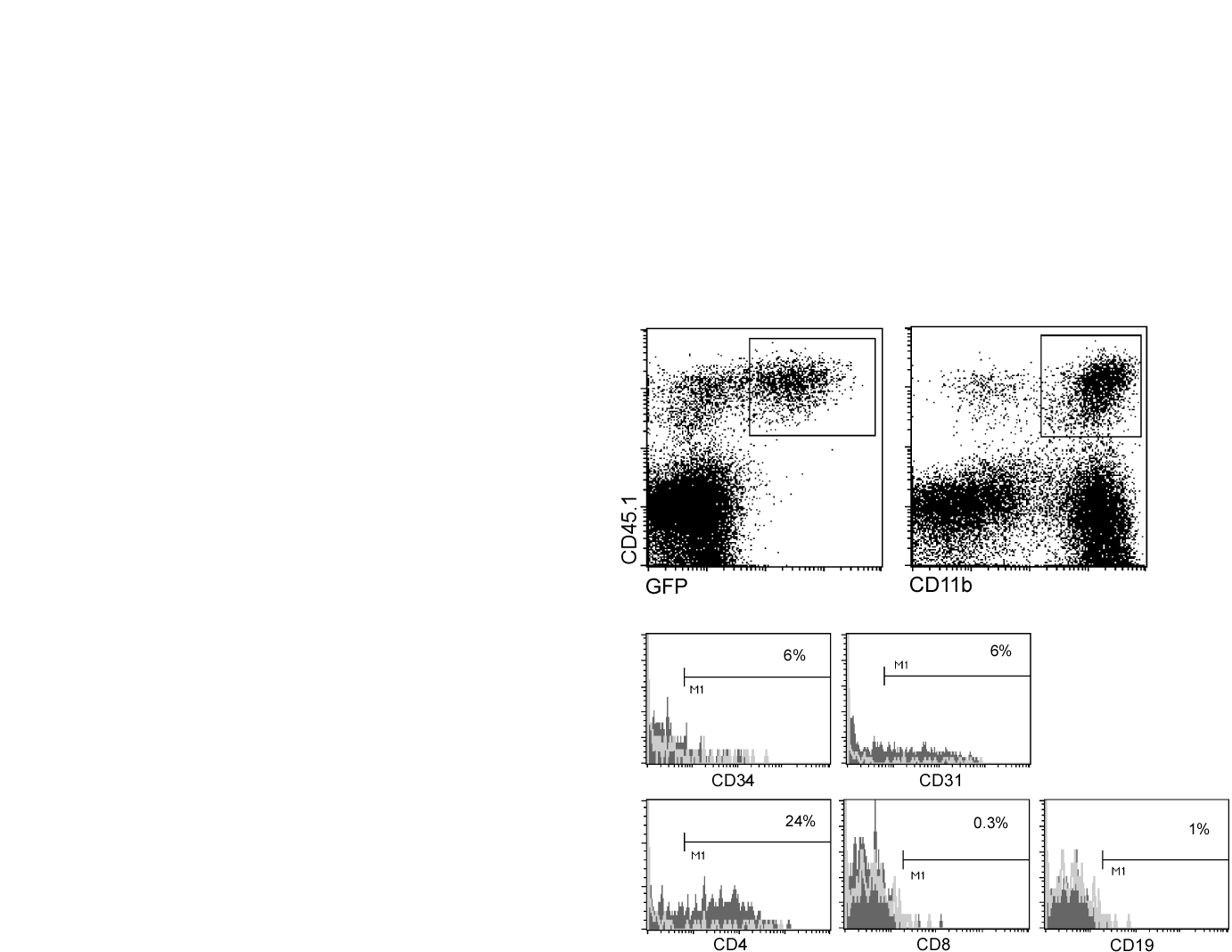

Supplement: Figure S4 — Monocyte-derived myeloid cells do not express lymphoid or progenitor markers. Representative flow cytometric plots of various lymphoid/ progenitor markers by the myeloid infiltrating cells (CD11b+/ Cx3cr1 GFP/+/CD45.1+) in injured [Cx3cr1 GFP/+ (CD45.1)>wt (CD45.2)] BM chimeras (isotype control, gray line). The percentage of the positive cells within the infiltrating myeloid population is indicated above each marker. (0.08 MB TIF) [file pmed.1000113.s004.tif]

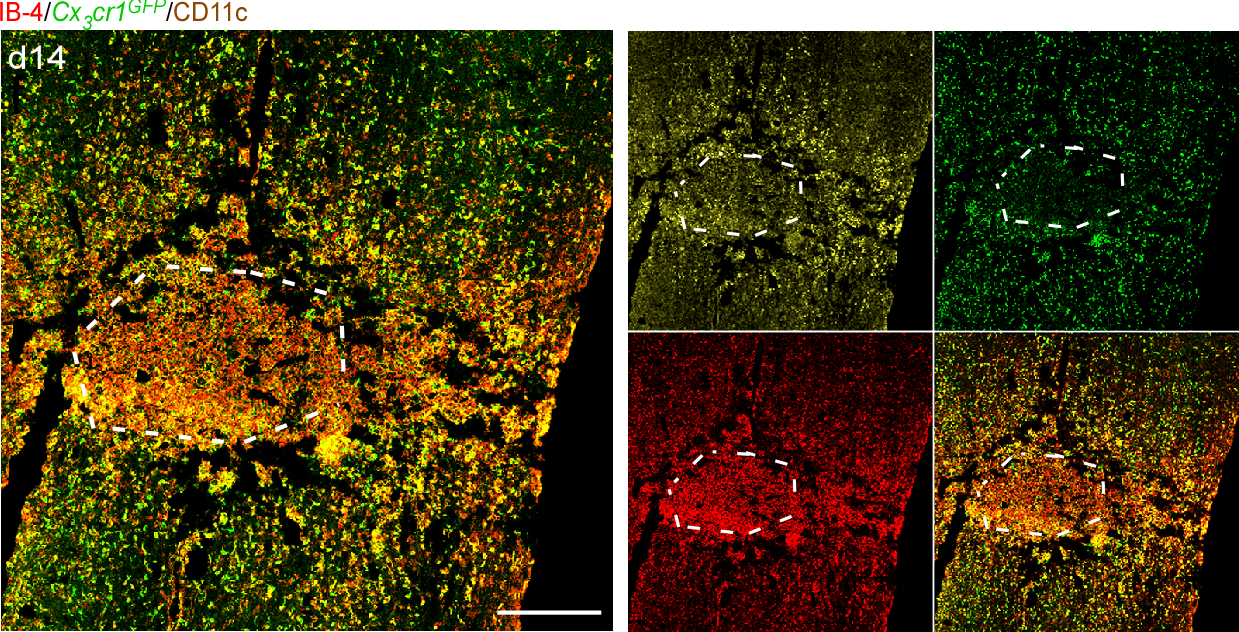

Supplement: Figure S5 — Expression of CD11c in the epicenter of the lesion site from d14 postinjury and onward. Longitudinal sections labeled for IB-4 (red), GFP (green) and CD11c (yellow), showing that, at later time points (d14 and onward), CD11c expression by the resident microglia (IB-4+/GFP− cells) was also observed at the epicenter of the lesion and not only at its margins. Right panel shows split images of the left panel. The dashed line demarcating the lesion site was determined according to GFAP immunoreactivity. Scale bar = 250 µm. (1.49 MB TIF) [file pmed.1000113.s005.tif]

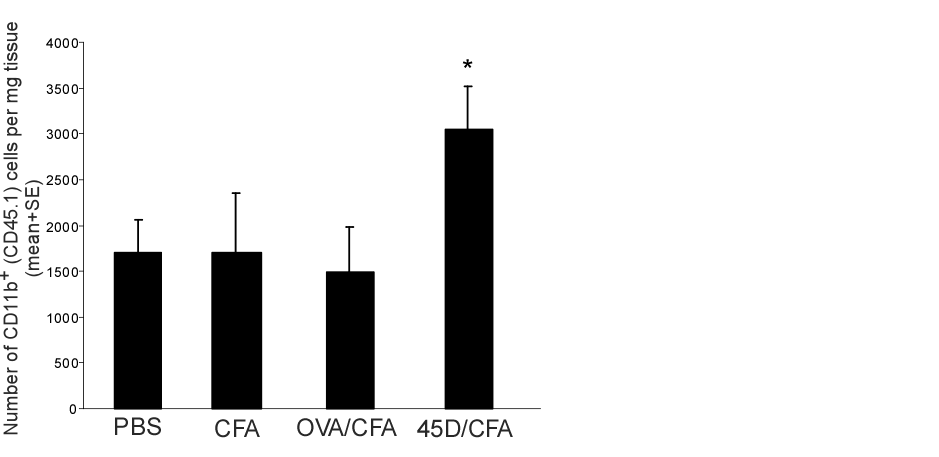

Supplement: Figure S6 — Vaccination with a CNS-specific antigen, rather than an irrelevant antigen, is required to augment infiltration of monocyte-derived macrophages to the injured spinal cord. [CD45.1>wt (CD45.2)] BM chimeras were vaccinated 7 d prior to spinal cord injury with: CNS specific antigen (45D/CFA), the irrelevant antigen ovalbumin (OVA/CFA), CFA, or PBS alone. The injured spinal cords were analyzed 1 wk after injury for the entrance of the infiltrating monocyte-derived MΦ (CD45.1+/CD11b+). Increased infiltration could be seen only in the mice that were vaccinated with CNS-specific antigen (ANOVA; F3,9 = 4.52; p = 0.03). Asterisk indicates significant differences between the indicated group to all other groups. y-Axis error bar represents SE. (0.02 MB TIF) [file pmed.1000113.s006.tif]

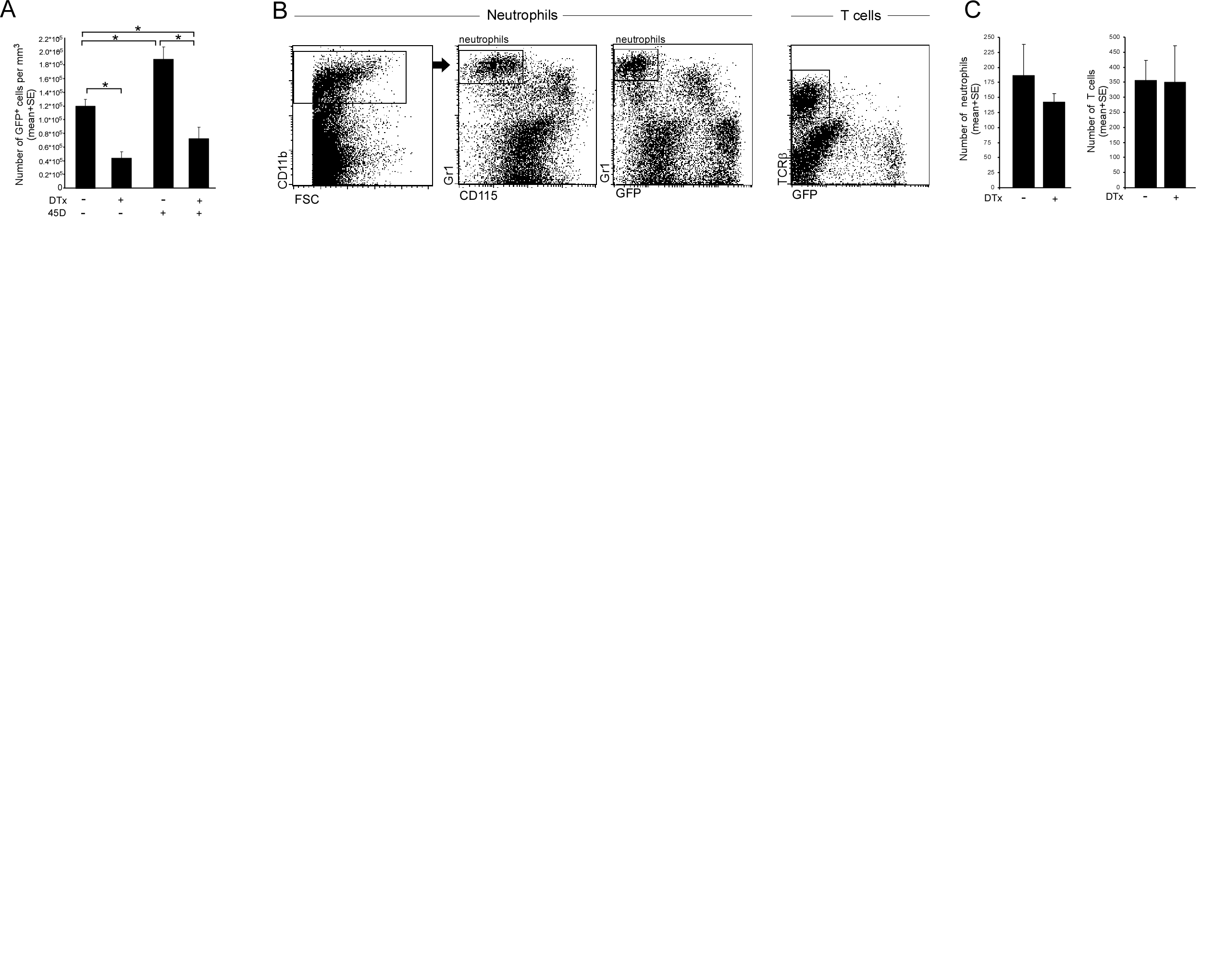

Supplement: Figure S7 — Diphtheria toxin depletes CD11c expressing monocyte-derived macrophages at the lesion area without significantly affecting infiltrates of other inflammatory cell types. Analysis at the injured site 14 d postinjury of [CD11c-DTR: Cx3cr1 GFP/+>wt] BM chimeras, treated without (w/o) or with DTx ablation. (A) Assessment of monocyte-derived MΦ; GFP+ cells, ANOVA; F3,6 = 21.5; p = 0.0013), and (B, C) of infiltrates of other inflammatory cell types. Neutrophils were identified by gating on the CD11b+/Gr1high and CD115−/GFP− population, while T cells were assessed by gating on TCRβ+ cells (Student's t-test; t = −0.5; df = 5; p = 0.65 and t = −0.04; df = 3; p = 0.97, respectively). y-Axis error bar represents SE. (0.12 MB TIF) [file pmed.1000113.s007.tif]

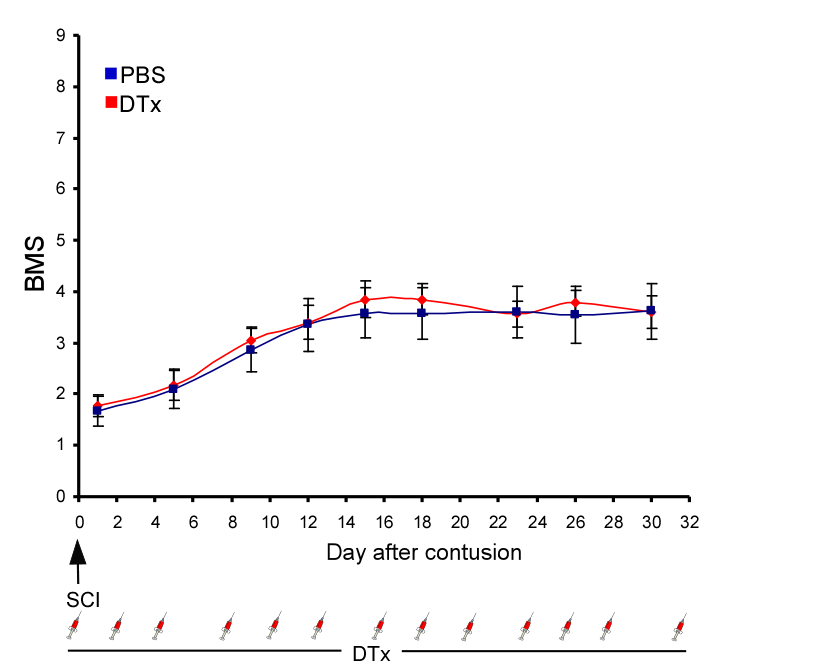

Supplement: Figure S8 — DTx treatment of wt mice that do not harbor the DTR transgene does not inhibit functional recovery from spinal cord injury. C57BL/6J mice were subjected to contusive SCI. Half of the animals were treated with DTx starting immediately after the injury. Locomotion was recorded at different time points following the injury, and is presented as the mean locomotor score (BMS) for each group. DTx administration to the nonchimeric (C57BL/6J) mice did not affect functional recovery following SCI (repeated measures ANOVA; F1,18 = 0.12; p = 0.73). y-Axis error bar represents SE. (0.03 MB TIF) [file pmed.1000113.s008.tif]

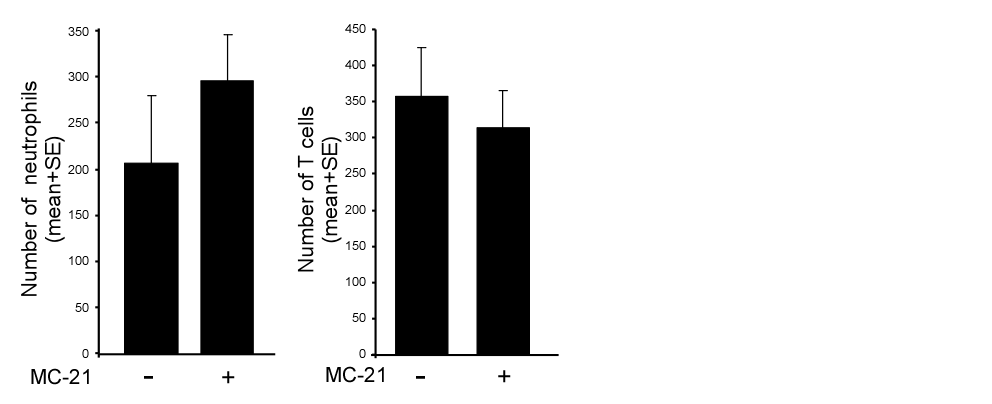

Supplement: Figure S9 — MC-21 treatment has no significant effect on other inflammatory cell types. Quantitative analysis 14 d postinjury of neutrophils and T cells at the injured site of [Cx3cr1 GFP/+>wt] BM chimeras, without (w/o) or with MC-21 treatment. Neutrophils were identified by gating on CD11b+/Gr1high and CD115−/GFP− population, while T cells were assessed by gating on TCRβ+ cells (Student's t-test; t = −0.85; df = 5; p = 0.43 and t = 0.5; df = 5; p = 0.63, respectively). y-Axis error bar represents SE. (0.02 MB TIF) [file pmed.1000113.s009.tif]

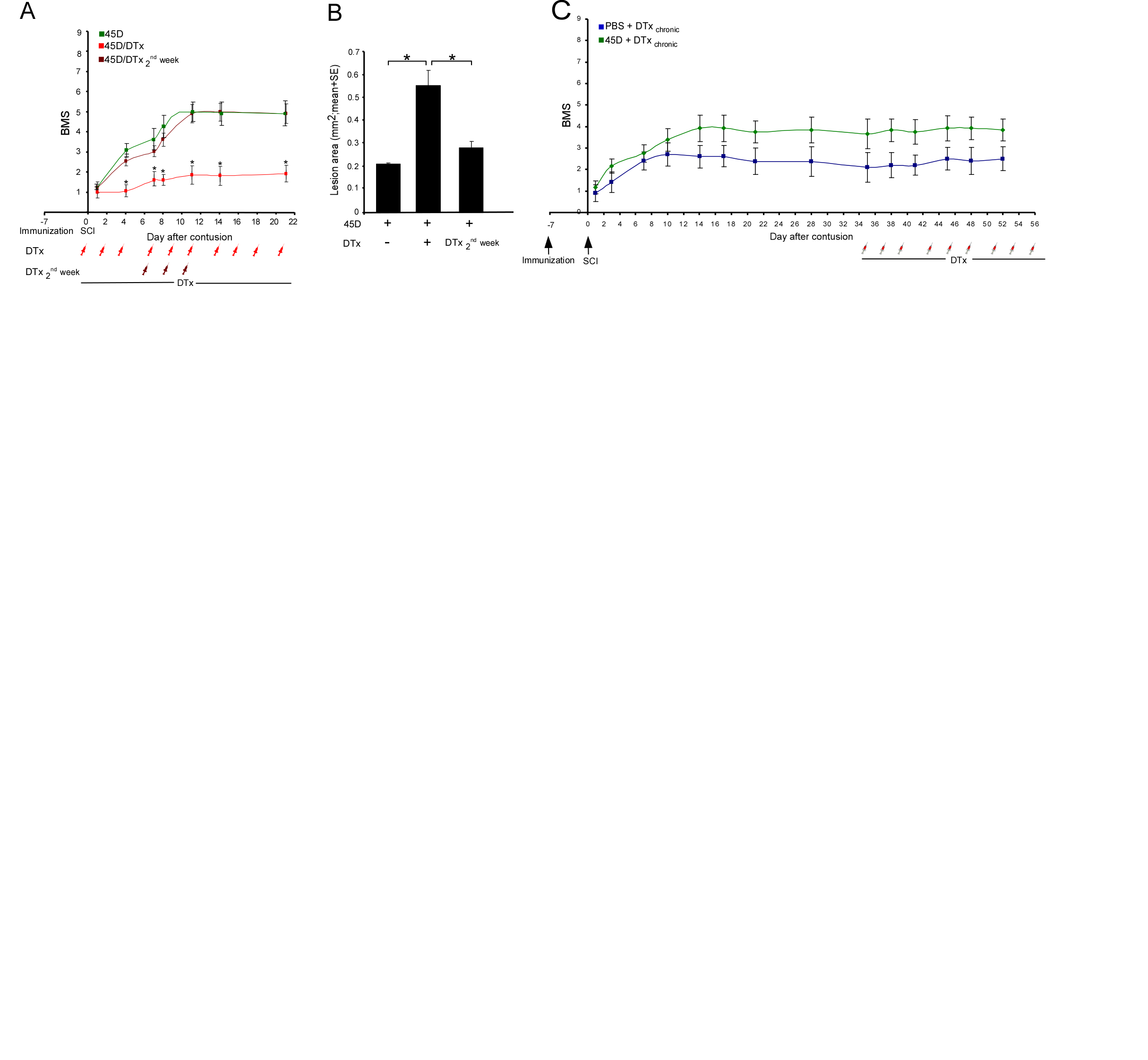

Supplement: Figure S10 — Ablation of monocyte-derived macrophages in the second week or at the chronic phase following spinal cord injury does not affect recovery. (A, B) [CD11c-DTR: Cx3cr1 GFP/+>wt] BM chimeras were vaccinated with 45D 7 d before SCI, and were treated with DTx during the second (2nd) week, along the entire period of recovery, or remained without DTx treatment. (A) Mean locomotor score (BMS) for each group as a function of time postinjury, showing that infiltrating monocyte-derived MΦ ablation does not affect recovery when it is carried out during the second week (repeated measures ANOVA; F[between groups]2,26 = 16.22; p = 0.0001). (B) Quantitative analysis of the size of the injury site as a function of treatment with DTx (ANOVA; F2,10 = 16.34; p = 0.007). (C) After verifying that monocyte-derived MΦ (GFP+) expressing CD11c were still found at the injury site 1 mo after injury, DTx was administered to immunized and nonimmunized mice DTx by repeated injections starting from 1 mo postinjury, when the animals had already reached plateau levels of recovery. The ablation of infiltrating monocyte-derived MΦ by DTx application at this late chronic stage had no effect on locomotor ability. Asterisks denote statistically significant differences between the indicated groups in (B) and compared to the relevant controls in (A). y-Axis error bar represents SE. (0.11 MB TIF) [file pmed.1000113.s010.tif]

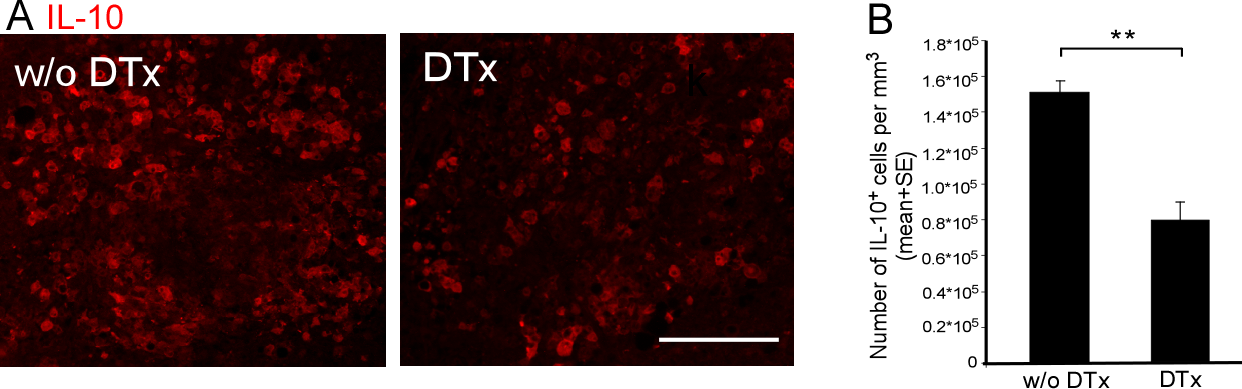

Supplement: Figure S11 — Ablation of monocyte-derived macrophages results in reduction of IL-10 levels in the injured site. (A, B) The levels of IL-10 at the injury site of [CD11c-DTR: Cx3cr1 GFP/+>wt] BM chimeras that were treated without (w/o) or with DTx were tested. (A) Representative micrographs of spinal cord sections labeled for IL-10, in the absence or presence of DTx treatment (scale bar = 100 µm). (B) Quantification of IL-10+ cells in the spinal cords with and without DTx treatment, based on immunohistochemical analysis (Student's t-test; t = −5.33; df = 6; p = 0.0018). y-Axis error bar represents SE. (0.34 MB TIF) [file pmed.1000113.s011.tif]

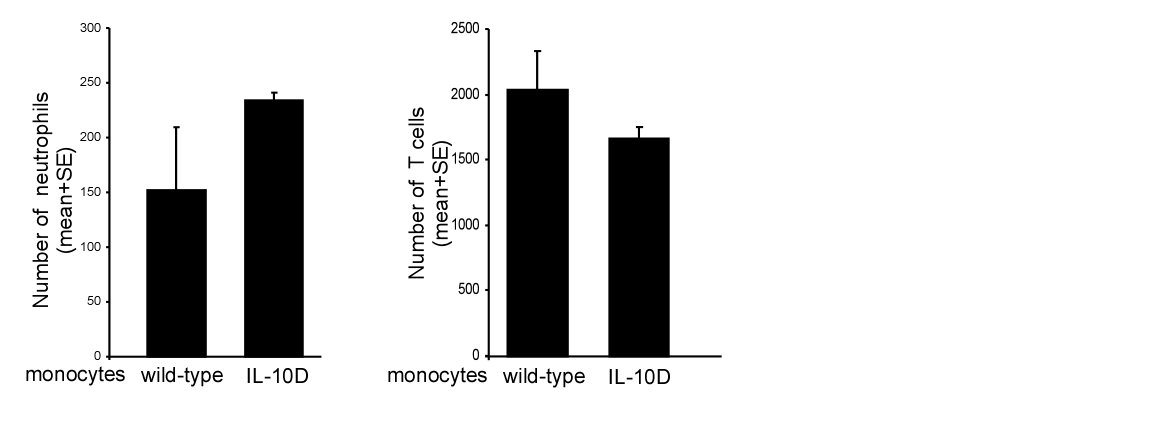

Supplement: Figure S12 — IL-10 deficiency in the infiltrating monocytes has no significant effect on other inflammatory infiltrates. Quantitative analysis 14 d postinjury of neutrophils and T cells at the injury site of [CD11c-DTR:Cx3cr1 GFP/+>wt] BM chimeras treated with DTx in parallel to adoptive transfer of either wt or IL-10–deficient monocytes. Neutrophils were identified by gating on the CD11b+/Gr1high and CD115−/GFP− population, while T cells were assessed by gating on TCRβ+ cells (Student's t-test; t = 2.2; df = 4; p = 0.11 and t = −1.0; df = 6; p = 0.35, respectively). y-Axis error bar represents SE. (0.03 MB TIF) [file pmed.1000113.s012.tif]
